# Progression of the faecal microbiome in preweaning dairy calves that develop cryptosporidiosis

**DOI:** 10.1186/s42523-024-00352-1

**Published:** 2025-01-06

**Authors:** M. F. Hares, B. E. Griffiths, L. Barningham, E. E. Vamos, R. Gregory, J. S. Duncan, G. Oikonomou, C. J. Stewart, J. L. Coombes

**Affiliations:** 1 Infection Biology and Microbiomes, Institute of Infection, Veterinary and Ecological Sciences, University of Liverpool, iC2 Liverpool Science Park, Liverpool, L3 5RF UK; 2 Livestock and One Health, Institute of Infection, Veterinary and Ecological Sciences, University of Liverpool, Leahurst Campus, Neston, Wirral, CH64 7TE UK; 3 Centre for Genomic Research, University of Liverpool, Crown Street, Liverpool, L69 7ZB UK; 4 Translational and Clinical Research Institute, Faculty of Medical Sciences, Newcastle University, Newcastle, NE2 4HH UK; 5 School of Pharmacy and Life Sciences, Robert Gordon University, Garthdee Road, Aberdeen, AB10 7GJ UK

**Keywords:** *Cryptosporidium parvum*, Bovine cryptosporidiosis, Calf diarrhoea/diarrhea, Longitudinal, Faecal/fecal microbiome, 16S rRNA gene amplicon sequencing

## Abstract

**Background:**

Cryptosporidiosis is a diarrheal disease that commonly affects calves under 6 weeks old. The causative agent, *Cryptosporidium parvum*, has been associated with the abundance of specific taxa in the faecal microbiome during active infection. However, the long-term impact of these microbiome shifts, and potential effects on calf growth and health have not yet been explored in depth.

**Methods:**

Three hundred and forty-six (346) calves from three dairy farms had one faecal swab collected during the first week of life (W1). Thereafter, sampled calves were monitored for diarrhoeal disease and those that suffered a diarrhoea event were tested for *C. parvum* by lateral flow testing (LFT). Calves that experienced diarrhoea and tested positive for *C. parvum* by LFT were assigned to the *Cryptosporidium*-positive (*Cp*+) group (*n* = 32). Matched healthy (H) controls with no history of diarrhoea were selected from the remaining cohort (*n* = 33). The selected subset of calves (*n* = 65) was observed until weaning, collecting a faecal swab, at approximately Week 5 (W5) and Week 10 (W10) after birth, resulting in a total of 191 samples (W1; *n* = 65, W5; *n* = 64, W10; *n* = 62). 16S rRNA gene amplicon sequencing was performed on all extracted samples.

**Results:**

Analysis of the longitudinal microbiome showed significant changes in the microbial diversity and composition across all three time-points. Whilst *Firmicutes* were elevated in the *Cp*+ group at W5 compared to the H group, no other significant differences were detected between H and *Cp*+ groups. Whilst the core microbiota showed some taxa were exclusive to each group, the role of these taxa in health and disease has yet to be determined. Antibiotics were also found to have an impact on the relative abundance of some taxa. Though healthy calves received a significantly higher body condition score than *Cp*+ calves at W5, the difference did not reach significance at W10, suggesting that *Cp*+ calves may catch up to their healthy counterparts once the infection has resolved.

**Conclusions:**

The findings of this study illustrated the changes in the microbial diversity and composition during the preweaning period in dairy calves. The results also indicated that the faecal microbiome is not predictive of cryptosporidiosis and implied that cryptosporidiosis doesn’t cause long-term gut dysbiosis. This study furthered our understanding of the parasite-microbiome relationship and its impact on the bovine host.

**Supplementary Information:**

The online version contains supplementary material available at 10.1186/s42523-024-00352-1.

## Introduction

*Cryptosporidium parvum* is an apicomplexan protozoan and is the leading cause of calf diarrhoea, accounting for approximately 70% of all *Cryptosporidium* infections in cattle [1]. The parasite invades the small intestinal epithelium of cattle, usually within the first six weeks of life, and can cause clinical signs such as watery diarrhoea, weight loss, dehydration, and in especially severe cases, can be fatal. These signs not only compromise cattle health and welfare but also place a financial burden on the farmer. A recent study found that dairy calves infected with *C. parvum* between 3 days and 3 weeks of age incurred an average cost of €40 (~£35/$43) per calf in losses through mortality, labour costs, and veterinary treatment [2]. Recently, the first commercially available efficacious gp40 subunit vaccine against bovine cryptosporidiosis was developed [3]. Late-gestation cows that were vaccinated exhibited a strong immune response, and this protection was effectively transferred to newborn calves via colostrum [3]. Whilst this vaccine reduced the incidence, severity and duration of diarrhoea caused by *C. parvum* in calves, and increased weight gain and reduced the rate of mortality, it wasn’t able to prevent clinical symptoms completely [3]. The main pharmacological treatment options are limited to halofuginone, a coccidiostat with variable efficacy, and high toxicity [4].

Given the economic and welfare implications in conjunction with a lack of completely efficacious therapies, it is of paramount importance to explore alternative approaches for preventing and treating bovine cryptosporidiosis. The parasite colonises and replicates in the ileum of the small intestine, and therefore it is likely that the parasite interacts with, manipulates, and is influenced by the commensal microbes that inhabit the gut. There are a large number of studies that show how the calf gut microbiome develops from birth to weaning in healthy and diarrhoeic calves [5–15]. A couple of studies investigate the progression of the intestinal microbiota in healthy calves [13, 16]. These studies highlight the microbial variation that occurs along the gastrointestinal tract (GIT). For example, they reported that *Lactobacillus* and *Clostridium* were the most prevalent genera in the ileum, whilst *Prevotella* and *Faecalibacterium* had the highest prevalence in the colon compared to other regions of the GIT [16]. While intestinal microbiome studies show a more accurate picture of the microorganisms that may directly interact with pathogens in the gut, the collection of tissue and digesta samples requires the culling of animals and prevents follow-up sampling to study the microbiome development within the same animal. Faecal microbiome studies, however, provide the means for longitudinal study designs, and non-invasive sample collection. Although, it can only be assumed that the faecal microbiome is a proxy for the microbial diversity and composition of the intestinal microbiome, as they are not directly comparable [17].

Existing research that explicitly studies the associations between the calf microbiome and *C. parvum* infection is limited. A study that inspected the faecal microbiomes of calves that were experimentally infected with *C. parvum,* showed that infection was associated with a high prevalence of *Clostridium* spp. as well as other potentially pathogenic bacteria such as *Escherichia/Shigella*, *Listeria*, and *Campylobacter* spp. [7]. Some studies have demonstrated that the faecal microbiomes of calves with cryptosporidiosis have a significantly higher abundance of *Fusobacterium* compared to healthy calves [18–20]. Whilst the majority of studies focus on the microbiome during *C. parvum* infection, there is little research on the diversity and composition of the microbiome prior to and post-infection, leaving a gap in our knowledge of whether a particular microbiome may be predictive of cryptosporidiosis or whether *C. parvum* infection causes long-term gut dysbiosis if calves overcome infection. Using whole-genome shotgun sequencing, we previously found that whilst the diversity and composition of the microbiome did not predict infection, specific functional pathways of the microbiome were found to be significantly associated with a predisposition to *C. parvum* infection [21]. The aim of this study was to determine how the bacterial diversity and composition of the faecal microbiome in healthy and *Cryptosporidium*-positive calves changed between birth and weaning. The objective was to ascertain taxa which are associated with the pre-/post-infection or healthy faecal microbiome, which could further our understanding of the host-parasite-microbiome relationship, and therefore assist in the development of novel therapeutics.

## Materials and methods

### Ethics

The study was conducted following ethical approval by the University of Liverpool Research Ethics Committee (VREC927) and procedures regulated by the Animals (Scientific Procedures) Act were conducted under a UK Home Office License (P191F589B).

### Animals

Three hundred and forty-six (346) female Holstein dairy calves from three commercial dairy farms, based in North Wales and Cheshire, UK (Farm 1, 2, and 3), were enrolled on to the study during the first week of life. Calves had rectal swabs collected at three time-points; preweaning pre-*C. parvum* infection (Week 1; *n* = 65), then at preweaning post-*C. parvum* infection (Week 5 ± 2; *n* = 65), and during the weaning stage post-infection (Week 10 ± 2; *n* = 64); classified hereafter as W1, W5, W10, respectively. All calves were enrolled on the study within 2 months, with the collection of all samples taking place across a period of 4 months between July and November 2020. A subset of the Week 1 samples were used in a shotgun sequencing study to determine the taxonomic and functional aspects of the faecal microbiome that may be associated with a predisposition to cryptosporidiosis [21]. Calves were weaned at approximately 10 weeks old. Trimethoprim and sulfadiazine (2.5 mg trimethoprim/12.5 mg sulfadiazine/kg, Diatrim^®^, Dechra, UK), amoxicillin and clavulanic acid (7.0 mg amoxicillin/1.75 mg clavulanic acid/kg, Synulox™ RTU, Zoetis, UK), and tulathromycin (2.5 mg/kg, Draxxin^®^, Zoetis, UK) were antibiotics prescribed on Farm 2 only. Trimethoprim and sulfadiazine or amoxicillin and clavulanic acid were administered metaphylactically on Farm 2 after an earlier outbreak of *E. coli* diarrhoea on this farm. Some calves received a second dose of Trimethoprim and sulfadiazine between W1 and W5. Halofuginone lactate (100 µg/kg (body weight), once a day, for 7 consecutive days, Halocur^®^, MSD Animal Health, UK) was administered prophylactically (Farm 1 and 3) or sometimes therapeutically (Farm 2), and therefore treated calves were included in the study. The date of administration and type of treatment were recorded for all subjects.

All calves received a similar diet; cow colostrum was delivered within 24 hours of birth, followed by milk replacer. The calves were then weaned onto a typical cereal and hay-based diet. The veterinary team deemed the breed and farm management of the sample population of calves on all farms to be typical of the UK dairy calf population. All calves were initially housed in individual pens up to 2 weeks old, and then either moved into large indoor group (15–20 calves) pens (Farm 2 and 3) or calf hutches with two other calves (Farm 1). All calves were bedded on straw. Diarrhoeic calves were not isolated unless the infection was severe and were individually housed for treatment. Calf health was monitored throughout the study by body condition score (BCS), body weight in kilograms (measured by scales or weigh tape), faecal consistency scoring (0 = Normal, 1 = Semi-formed, pasty, 2 = Loose, but stays on top of bedding, 3 = Watery, sifts through bedding) and the Wisconsin (WI) scoring system to assess respiratory disease, to determine the overall health status of the calves [22, 23]. The serum total protein was quantified within the first week of life as an additional measure of calf health. Thoracic ultrasonography was employed to detect the severity of historic respiratory damage during the weaning stage (W10) and calves were assigned a lung score of 0–5 (0–1 = Normal, 2 = Lobular/patchy pneumonia, 3–5 = Severe lobar pneumonia − 1, 2 or ≥ 3 lobes affected). Calves did not display clinical signs of cryptosporidiosis at any of the sampling time-points. Diarrhoea events usually occurred between the W1 and W5 sampling, though one calf did experience an additional diarrhoea event between the W5 and W10 sampling. The study design and timeline for sampling, diarrhoea events, and treatment administration are presented in Additional file 1: Fig. S1-S2.

### Sample collection

Prior to the development of clinical signs of cryptosporidiosis, one faecal swab sample (Sterilin Regular Nylon Flocked Swabs 552 C, Scientific Laboratory Supplies, UK) was collected from each of the 346 calves during the first week of life (W1), and snap frozen on dry ice directly after the sampling. Shortly after the collection, samples were stored at – 80 °C until DNA extraction. Following W1 sampling, the calves were monitored by experienced veterinary clinicians using a faecal score (0–3) to determine the occurrence of diarrhoea events [23]. Calves that experienced a diarrhoea event after W1 sampling (faecal score of ≥ 2) were assessed for infectious agents using lateral flow testing (LFT) (MSD Rainbow Calf Scour Diagnostic Test, Farmacy, UK), to detect *Rotavirus*, *Coronavirus*, *E. coli* F5 (K99) and *C. parvum*. During the study period, 32 calves developed diarrhoea and tested positive for *C. parvum* on the LFT, two of which also tested positive for *Rotavirus*. Thirty-three (33) healthy matched controls were selected from the remaining sampled cohort. The healthy calves were selected on the basis that they showed no clinical signs of diarrhoeal disease during the whole sampling period, though calves with respiratory disease signs were included in the study. The control calves were matched to the *Cp*+ group by age, sex, farm and breed, and as closely matched for administration date and type of antibiotic treatment as possible. Selected calves were sampled again using available swabs at preweaning once diarrhoea events had resolved (W5; *n* = 65), and again during the weaning stage (W10; *n* = 64; one calf did not have a sample collected) (Sterilin Regular Nylon Flocked Swabs 552 C, Scientific Laboratory Supplies; COPAN FLOQSwabs COPA961C, VWR, UK; Oropharyngeal Specimen Collection Swab MD300235, Medline Scientific, UK). The selected calves that did not experience a diarrhoea event and remained healthy throughout the study were referred to as the Healthy (H) group (*n* = 33). Calves that experienced a diarrhoea event (faecal score of ≥ 2) and received a positive test result for *C. parvum* were referred to as the *Cryptosporidium*-positive (*Cp*+) group (*n* = 32). All samples were categorised according to health status and sample time-point (H1, H5, and H10; *Cp*+1, *Cp*+5, and *Cp*+10). Some W5 (*n* = 1) and W10 (*n* = 2) samples went missing in transit, resulting in a total of 191 samples (W1; *n* = 65, W5; *n* = 64, W10; *n* = 62) that were carried forward for extraction, sequencing, and analysis.

### DNA extraction

DNA extraction of faecal swab samples (*n* = 191) was carried out using the DNeasy^®^ PowerLyzer^®^ Powersoil^®^ Kit (QIAGEN, UK) as per the manufacturer’s instructions with the following exceptions. Faecal swabs were transferred into Powerbead tubes, and sterilised scissors (submerged in 100% ethanol and flamed in a Bunsen burner between samples) were used to remove excess swab applicator. 500 µL of Powerbead solution was combined with C1 solution in the Powerbead tubes, which were arranged securely in a 24-tube adaptor on a Vortex-Genie™ 2 (Scientific Industries Inc., USA) for 15 min at speed 7.5. Solutions C2 and C3 were combined 1:1 and 300 µL per sample was mixed with the sample supernatant. This mixture was incubated at 4 °C for 5 min. 50 µL of Solution C6 was added directly to the centre of the white filter membrane to elute DNA. DNA was quantified by NanoDrop™ One (ThermoFisher Scientific Inc., UK), and Qubit™ dsDNA Quantitation, Broad Range Kit and Qubit™ 3.0 (Invitrogen™, ThermoFisher Scientific, UK) and the optimum qPCR cycle length of 30 was determined by qPCR. All negative extraction and negative swab controls contained negligible quantities of DNA and so only one negative extraction control was selected to be carried forward for sequencing. DNA samples were stored at – 80 °C until sequencing.

### Amplicon library preparation

One hundred and ninety-one (191) gDNA samples and controls (One negative extraction control, three negative PCR plate controls, and three ZymoBIOMICS microbial community standard positive PCR plate controls) underwent PCR using previously described primers to amplify the V4 hypervariable region of the 16S rRNA gene [24]:

F: 5’ACACTCTTTCCCTACACGACGCTCTTCCGATCTNNNNNGTGCCAGCMGCCGCGGTAA3’.

R: 5’GTGACTGGAGTTCAGACGTGTGCTCTTCCGATCTGGACTACHVGGGTWTCTAAT3’.

Five microlitres of DNA entered a first round PCR with cycling conditions that consisted of 20 s at 95 °C, 15 s at 65 °C, 30 s at 70 °C for 10 cycles then a 5-minute extension at 72 °C.

The primer design incorporated a recognition sequence to allow a secondary nested PCR process. Samples were first purified with AMPure XP Bead-Based Reagent (Beckman Coulter Ltd., UK) before entering the second PCR. The second PCR was performed to incorporate Illumina^®^ adapter sequences for sequencing of samples on the Illumina^®^ sequencing platforms. Barcodes for sample identification were also included at this point. Eight forward primers (i5) and twelve reverse primers (i7) each contained a separate barcode creating up to 96 different combinations. The barcode sequences were the same as those described in the Illumina^®^ Nextera protocol.

The general sequences of the forward and reverse primers are illustrated below. The 10 bp barcode is underlined.

N501 F: 5’AATGATACGGCGACCACCGAGATCTACACTAGATCGCATACACTCTTTCCCTACACGACGCTC3’.

N701 R: 5’CAAGCAGAAGACGGCATACGAGATTCGCCTTACTGTGACTGGAGTTCAGACGTGTGCTC3’.

Twenty cycles of second round PCR were performed using the same conditions as above for a total of 30 cycles. Samples were purified using AMPure XP Bead-Based Reagent before being quantified using the Qubit dsDNA Quantitation High Sensitivity Kit (Invitrogen™, ThermoFisher Scientific Inc., UK) and assessed using the Fragment Analyzer High Sensitivity NGS Fragment Kit (1–6000 bp) (Agilent Technologies LDA UK Ltd., UK). Successfully generated amplicon libraries were taken forward. These final libraries were pooled in equimolar amounts using the Qubit™ 3.0 (Invitrogen™, Thermo Fisher Scientific Inc., UK) and Fragment Analyzer (Agilent Technologies LDA UK Ltd., UK) data and cleaned up with AMPure XP Bead-Based Reagent at a 1:1 ratio. The quantity and quality of the pool was assessed by the Bioanalyzer High Sensitivity DNA Kit (Agilent, USA) and subsequently by qPCR using the KAPA Illumina^®^ Library Quantification Kit (Roche Diagnostics Ltd., UK) on a LightCycler LC480II (Roche Diagnostics Ltd., UK) according to manufacturer’s instructions. Briefly, a 10 µl PCR reaction (performed in triplicate for each pooled library) was prepared on ice with 8 µl SYBR Green I Master Mix (Roche Diagnostics Ltd., UK) and 2 µl diluted pooled DNA (1:1000 to 1:100,000 depending on the initial concentration determined by the Qubit™ dsDNA HS Assay Kit (Invitrogen™, Thermo Fisher Scientific Inc., UK)). PCR thermal cycling conditions consisted of 95 °C for 5 min, 35 cycles of 95 °C for 30 s and 60 °C for 45 s, melt curve analysis to 95 °C and cooling at 37 °C.

### Sequencing

Following calculation of the molarity using the qPCR data, template DNA was diluted to 12 pM and denatured for 5 min at room temperature using freshly diluted 0.2 N sodium hydroxide (NaOH) (Merck Life Science UK Limited, UK) and the reaction was subsequently terminated by the addition of HT1 buffer (Illumina^®^, San Diego, USA). To improve sequencing quality control, 15% PhiX (Illumina^®^, USA) was spiked in. The libraries were sequenced on the MiSeq platform (Illumina^®^, San Diego, USA), generating 2 × 250 bp and 2 × 300 bp paired-end reads. Raw data were uploaded to the National Center for Biotechnology Information Sequence Read Archive (BioProject PRJEB70717).

### Sequence processing

The raw fastq files generated by the 16S rRNA gene sequencing were trimmed for the presence of Illumina^®^ adapter sequences using Cutadapt version 1.2.1 [25]. The option -O 3 was used, so that the 3’ end of any reads which matched the adapter sequence for three or more base pairs were trimmed. The reads were further trimmed using Sickle version 1.2 with a minimum window quality score of 20 [26]. Reads shorter than 15 base pairs after trimming were removed. If only a single read pair passed this filter, it was included in the R0 file. Statistics showing the number of reads and read length per sample were generated using fastq-stats from EAUtils (Additional file 1: Fig. S3-S4) [27].

After trimming, R1 and R2 compressed fastq files (.gz) were processed in mothur 1.47.0 using a custom pipeline based on the MiSeq SOP protocol [28, 29]. Contigs were generated and primers removed before aligning the sequences with the SILVA version 132 reference alignment. Sequences were screened and filtered, and unique sequences identified. These unique sequences were pre-clustered, and chimeras were removed before assignment to their taxonomy using the RDP (PDS) training set version 18 reference sequences. Any sequences from chloroplast, mitochondria, *Archaea*, *Eukaryota* or unknown lineages were removed, and remaining sequences were assigned to operational taxonomic units (OTUs). OTU and consensus taxonomy tables were constructed, as well as a tree file of representative OTU sequences using the get.oturep command. The full breakdown of the mothur pipeline is presented in Additional file 2.

### Statistical analysis

All analysis of the resulting OTU and taxonomy tables was carried out in mothur 1.47.0 and RStudio with R version 4.3.1, using the following R packages: vegan 2.6-4, phyloseq 1.44.0, pairwiseAdonis 0.4.1, VennDiagram 1.7.3, emmeans 1.8.9, and MaAsLin2 1.15.1 [28–37]. The distribution of sample sequencing depth was determined (Additional file 1: Fig. S5). The sample containing the fewest number of reads (19,792 reads) was used to establish a cut-off of 19,000 reads to allow for inclusion of 100% of samples after rarefaction. All sample sequences were rarefied to 19,000 reads per sample to account for variation in read depth before commencing further analysis (Additional file 1: Fig. S6).

Alpha diversity was quantified by means of observed OTUs and the Shannon index in mothur. The resulting files were plotted in R as bar plots and scatterplots with local regression curves. Significant differences between subject parameters were determined using a pairwise Wilcoxon test with Benjamini-Hochberg correction for the bar plots and LOESS (locally estimated scatterplot smoothing) was implemented for the scatter plots to highlight trends in the data. The estimated marginal means were calculated to determine if there was a significant difference in alpha diversity between the H and *Cp*+ groups across the days of sampling.

Beta diversity was calculated by Bray-Curtis and Jaccard distance matrices in mothur using the dist.shared command. PCoA coordinates were produced by the pcoa commands in mothur and plotted in an ordination plot using vegan and ggplot2. Significant differences in beta diversity between H and *Cp*+ groups and sample time-points were determined by a pairwise PERMANOVA test with Benjamini-Hochberg correction using the pairwise.adonis2 function.

Taxonomic relative abundances at phylum and genus level were determined in R using phyloseq. The OTU table was rarefied to 19,000 reads per sample and parsed by taxonomic rank. Relative abundances of phyla and genera were plotted in stacked bar charts and the top 30 genera were plotted in individual boxplots. Significant differential abundances of phyla and genera were determined using MaAsLin2. Confounding variables such as farm, and routine treatments were included as fixed effects in addition to the parameters of interest; disease status and sample time-point. Samples from Farm 2 and Farm 3 were analysed separately and together to determine the farm effect and the impact of antibiotic use on Farm 2. Sample time-point was included as a continuous variable (day of sampling) to account for intra-time-point variation, and so the model would consider the three time-points longitudinally (Additional files 3 and 5). Study ID was used as a random effect to account for repeated measures from the same animal at different time-points. Pairwise multivariate comparisons were conducted using the Benjamini-Hochberg correction to recalculate *q*-values to correct for multiple testing to determine any significant differences in taxa between H and *Cp*+ groups within each sample time-point. *Q*-values that were ≤ 0.25 were considered significant (Additional files 4 and 6).

Daily live weight gain (DLWG; kg day^− 1^) for Farm 2, and body condition score, total WI score, and lung scores for all farms were plotted using ggplot2. A pairwise t-test or Wilcoxon test with Benjamini-Hochberg correction was performed to ascertain any significant differences between the H and *Cp*+ groups for each of these variables. All statistical analysis is presented in Additional file 2.

## Results

### *Cryptosporidium parvum* infection has a negligible effect on the diversity of the pre- and post-infection faecal microbiome

**Fig. 1:**
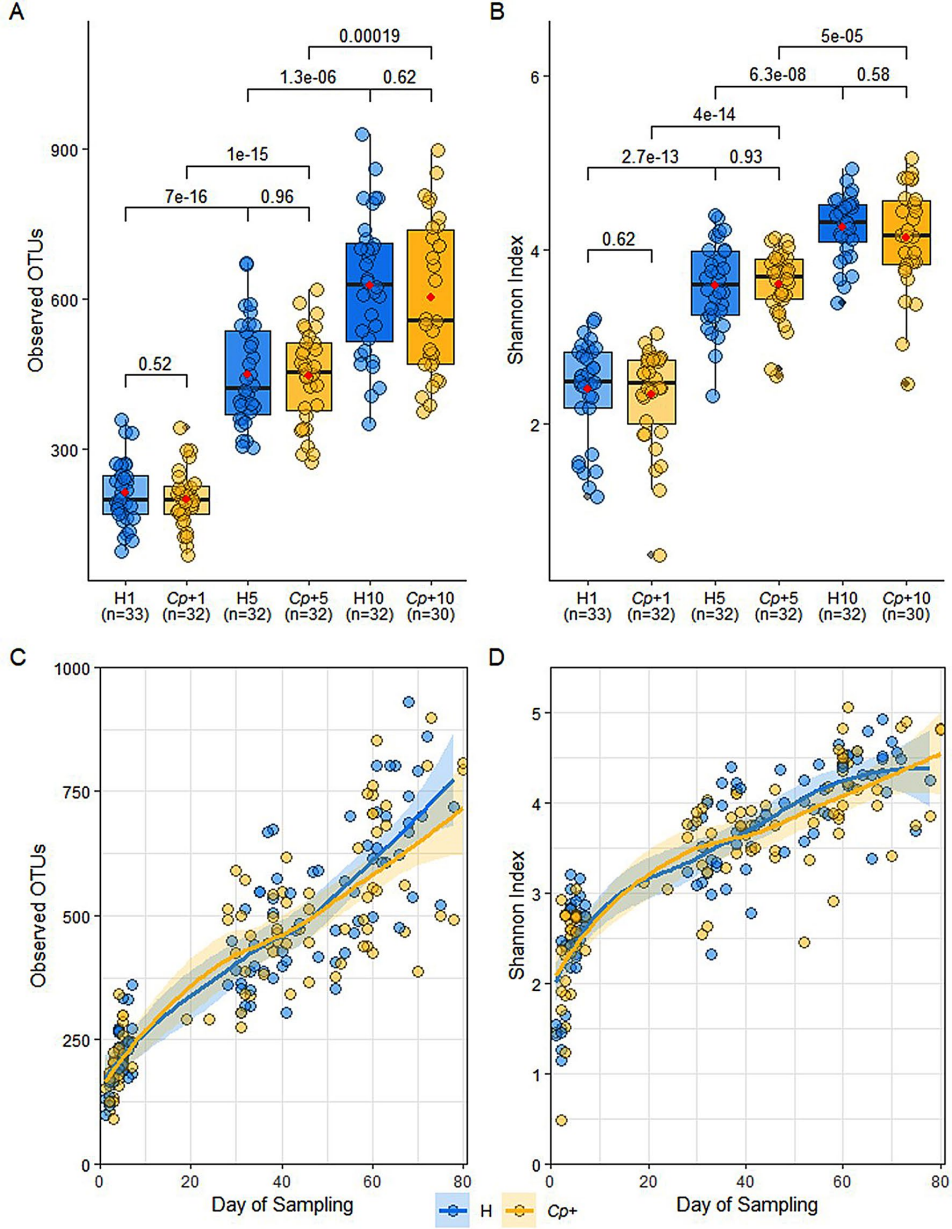
Alpha diversity increased with age but was not influenced by *Cryptosporidium parvum*. **A** Observed OTUs between healthy control and *Cryptosporidium-*positive groups at Week 1, 5, and 10. A pairwise Wilcoxon test shows differences in observed OTUs between time-points. The red dots indicate the mean. **B** Shannon diversity between healthy control and *Cryptosporidium-*positive groups at Week 1, 5, and 10. A pairwise Wilcoxon test shows differences in Shannon indices between time-points. The red dots indicate the mean. **C** Local regression with locally estimated scatterplot smoothing (LOESS) of observed OTUs between healthy control and *Cryptosporidium-*positive groups stratified by day of sampling shows within time-point variation. **D** Local regression with LOESS of Shannon diversity between healthy control and *Cryptosporidium-*positive groups stratified by day of sampling shows within time-point variation

16S rRNA gene amplicon sequencing was performed in order to ascertain how the diversity of the calf faecal microbiome changed between birth and weaning in calves that developed cryptosporidiosis compared to healthy controls. There was a significant increase across time-points in both observed OTUs (Median ± SE: W1; 199 ± 7, W5; 445 ± 12, W10; 619 ± 19, W1 vs. W5; Wilcoxon, *p* < 2.22E-16, W1 vs. W10; Wilcoxon, *p* < 2.22E-16, W5 vs. W10; Wilcoxon, *p* = 1.40E-09) and Shannon diversity (Median ± SE: W1; 2.49 ± 0.07, W5; 3.66 ± 0.05, W10; 4.27 ± 0.06, W1 vs. W5; Wilcoxon, *p* < 2.22e-16, W1 vs. W10; Wilcoxon, *p* < 2.22e-16, W5 vs. W10; Wilcoxon, *p* = 7.90e-11). However, there was no significant difference in the mean observed OTUs or Shannon diversity between H and *Cp*+ groups within any of the time-points (Fig. 1A-B; H1 vs. *Cp*+1; Wilcoxon, *p* = 0.45, H5 vs. *Cp*+5; Wilcoxon, *p* = 0.96, H10 vs. *Cp*+10; Wilcoxon, *p* = 0.56). The H and *Cp*+ groups were stratified by day of sampling to consider intra-group variation and plotted as a local regression (Fig. 1C-D). Overall, there was no significant difference in the estimated marginal means of observed OTUs (*p* = 0.60; Fig. 1C) or Shannon diversity (*p* = 0.92; Fig. 1D). When the samples were stratified by farm, no significant difference was observed between the H and *Cp*+ groups on Farm 2 or Farm 3 (Additional file 1: Fig. S7-S8).

**Fig. 2:**
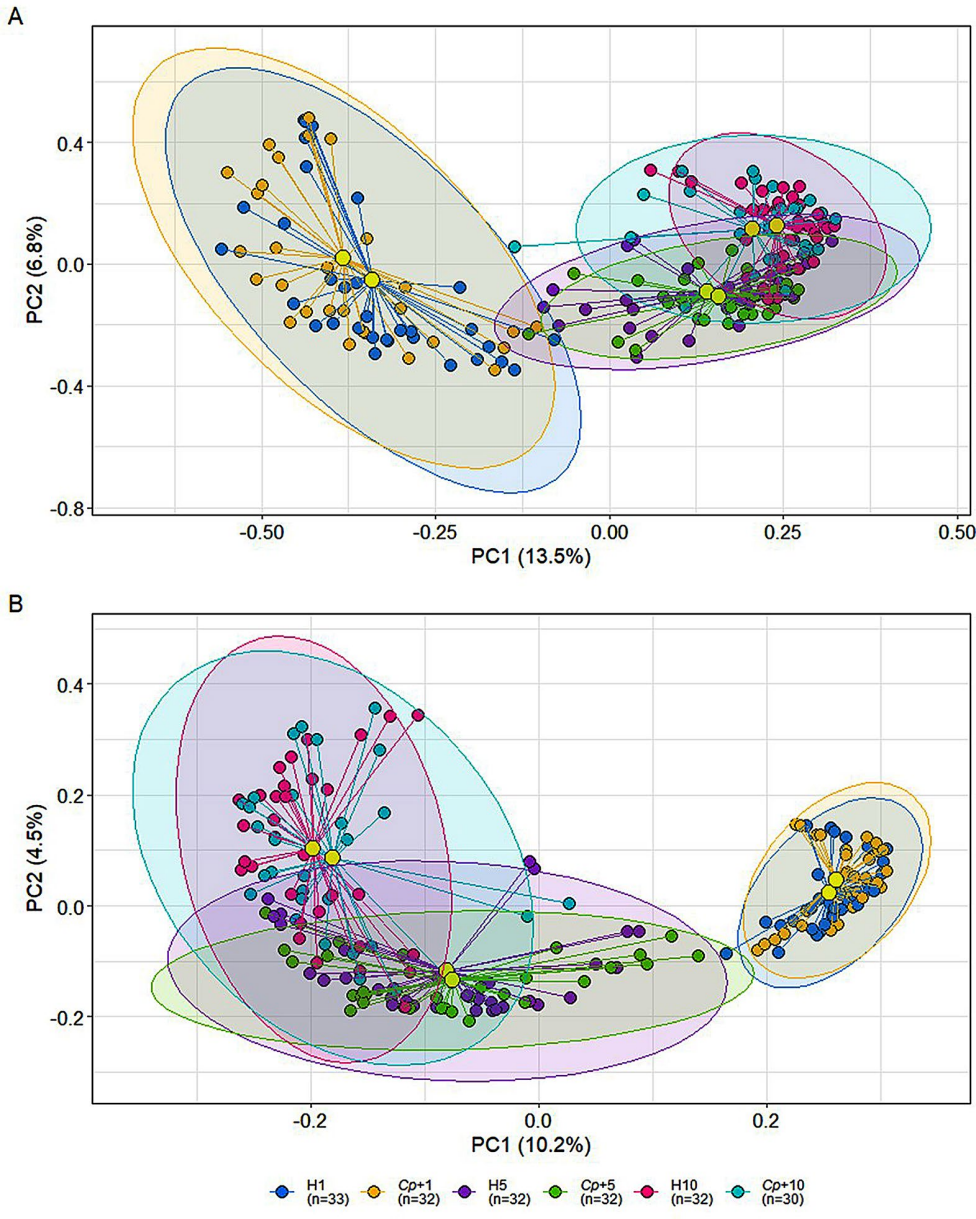
Beta diversity altered with age but was not impacted by *Cryptosporidium parvum*. **A** Weighted Bray-Curtis PCoA plot shows similarity between healthy control and *Cryptosporidium-*positive groups at Week 1, 5, and 10, and dissimilarity between sample time-points (pairwise PERMANOVA: H1 *vs. Cp*+1, *p* = 0.49; H5 *vs. Cp*+5, *p* = 0.40; H10 *vs. Cp*+10, *p* = 0.89; W1 vs. W5 vs. W10, *p* = 0.001). **B** Unweighted Jaccard PCoA plot displays similarity between healthy control and *Cryptosporidium-*positive groups at Week 1, 5, and 10, and dissimilarity between sample time-points (pairwise PERMANOVA: H1 vs. *Cp*+1; *p* = 0.20, H5 vs. *Cp*+5; *p* = 0.69, H10 vs. *Cp*+10; *p* = 0.90; W1 vs. W5 vs. W10, *p* = 0.001)

Assessment of beta diversity using Bray-Curtis PCoA showed that faecal microbiome samples were clustered together more closely over time (Fig. 2A), implying that there was a reduction in variance within each community. In contrast, the Jaccard PCoA showed an increase in variance over time (Fig. 2B). However, the Jaccard index is only a measure of presence or absence of OTUs and does not take abundance into account, therefore, it is not biased towards the most abundant OTUs. The increase in variance over time in the Jaccard plot may be due to the increase in the number of different OTUs and greater variance in the rarer OTUs between samples. The Bray-Curtis plot shows a reduction in variance as it takes abundance into account and therefore, the most abundant OTUs appear to homogenise across the samples over time. No statistically significant difference in variance was found between H and *Cp*+ groups within any time-point in either the Bray-Curtis or Jaccard metrics (Fig. 2A-B; pairwise PERMANOVA, *p* > 0.05). However, there was a statistically significant difference in both measures of beta diversity across the three time-points (Fig. 2A-B; pairwise PERMANOVA, *p* = 0.001). As W5 and W10 samples were collected with a ± 2 weeks range the post-infection samples follow a more continuous sampling pattern which is reflected in Fig. 1C-D. As a result, Fig. 2A-B shows that the variance of some of the W5 samples are more similar to the W1 samples and some W5 are more similar to the W10 samples. These trends remained the same when Farm 2 and Farm 3 were analysed separately (Additional file 1: Fig. S9). *C. parvum* infection had little impact on the pre- or post-infection community diversity, whilst the greatest difference in variance was observed between the different time-points. The reduction in Bray-Curtis variance over time is consistent with other studies that have investigated the development of the calf faecal microbiome.

### *Cryptosporidium parvum* has minimal impact on the calf pre- and post-infection faecal microbiome composition

**Fig. 3:**
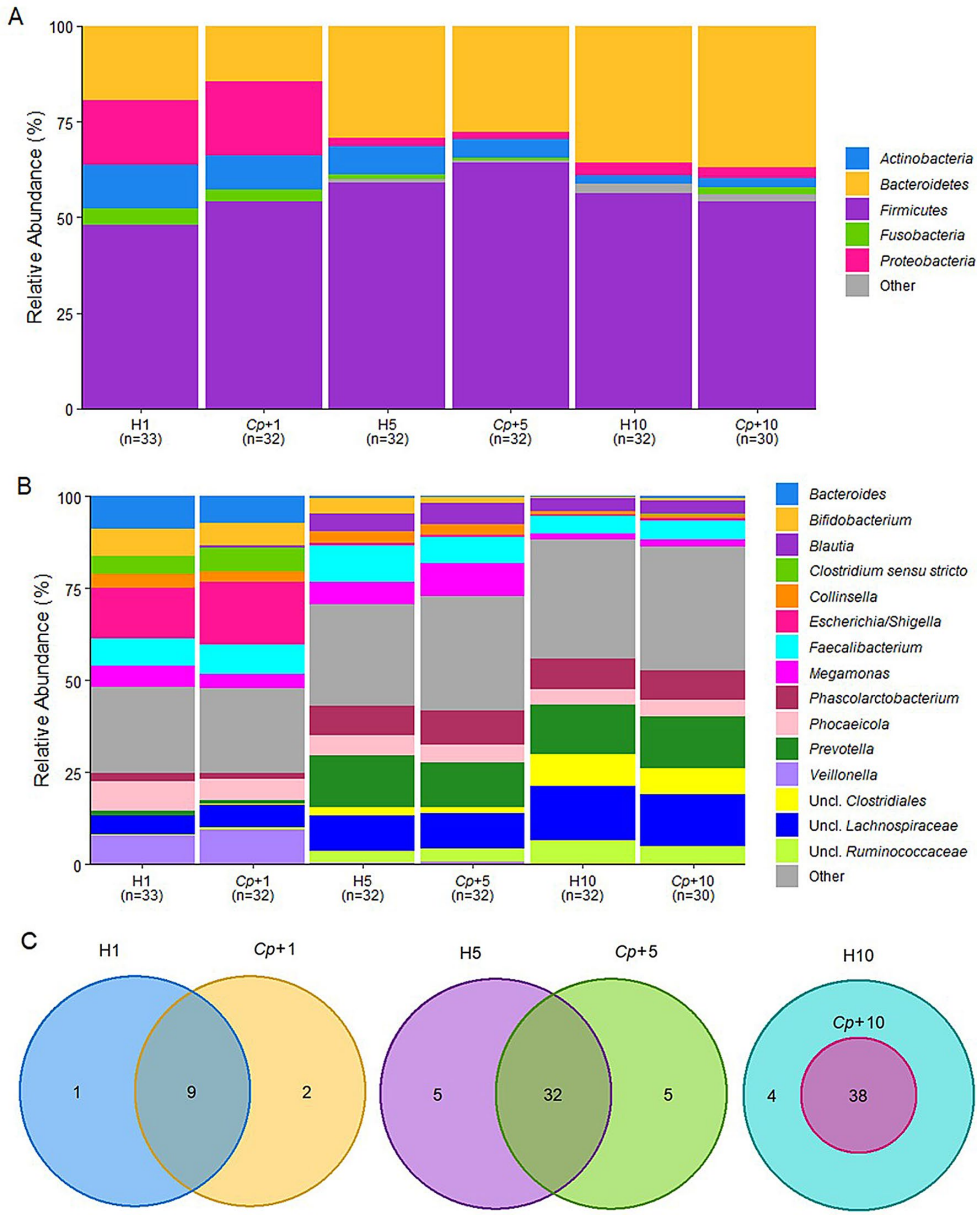
The pre-/post-infection taxonomic composition and core microbiome were minimally impacted by *Cryptosporidium parvum* infection. **A** Top 5 most abundant phyla at Week 1, 5, and 10 in healthy control and *Cryptosporidium-*positive groups. **B** Top 15 most abundant genera at Week 1, 5, and 10 in healthy control and *Cryptosporidium-*positive groups. **C** Venn diagrams of the core microbiota in healthy control and *Cryptosporidium-*positive groups at Week 1, 5, and 10 time-points. Genera were detected at 0.01% abundance in at least 80% of the samples

The faecal microbiome of calves that developed cryptosporidiosis or remained healthy was investigated to determine the impact of infection on the bacterial composition at three time-points between birth and weaning. While the most influential variable contributing to the differences in composition of the faecal microbiome was the day of sampling, the relative abundance of the phylum, *Firmicutes*, was found to be significantly higher in the *Cp*+ group compared to the H group at W5 (64.2% vs. 59.1%; *q* = 1.24E-01, Additional file 4). However, the levels of *Firmicutes* between H and *Cp*+ groups were no longer significantly different by W10 (Additional file 4). The two most abundant phyla in W1 were *Firmicutes* (50.9%) and *Proteobacteria* (18.0%) and the two most abundant phyla in W5 and W10 were *Firmicutes* (W5; 61.6%, W10; 55.2%) and *Bacteroidetes* (W5; 28.6%, W10; 36.4%). At the phylum level, *Firmicutes* (*q* = 2.20E-01) and *Bacteroidetes* (*q* = 4.92E-15) significantly increased in relative abundance over time (Fig. 3A, Additional file 3); whilst *Proteobacteria* (*q* = 6.30E-08), *Fusobacteria* (*q* = 5.86E-02), and *Actinobacteria* (*q* = 2.03E-07) significantly declined in relative abundance over time (Fig. 3A, Additional file 3). When analysed separately and together neither Farm 2 or Farm 3 showed any significant differences in phyla composition between the H and *Cp*+ groups within each time-point (Additional file 5–10).

**Fig. 4:**
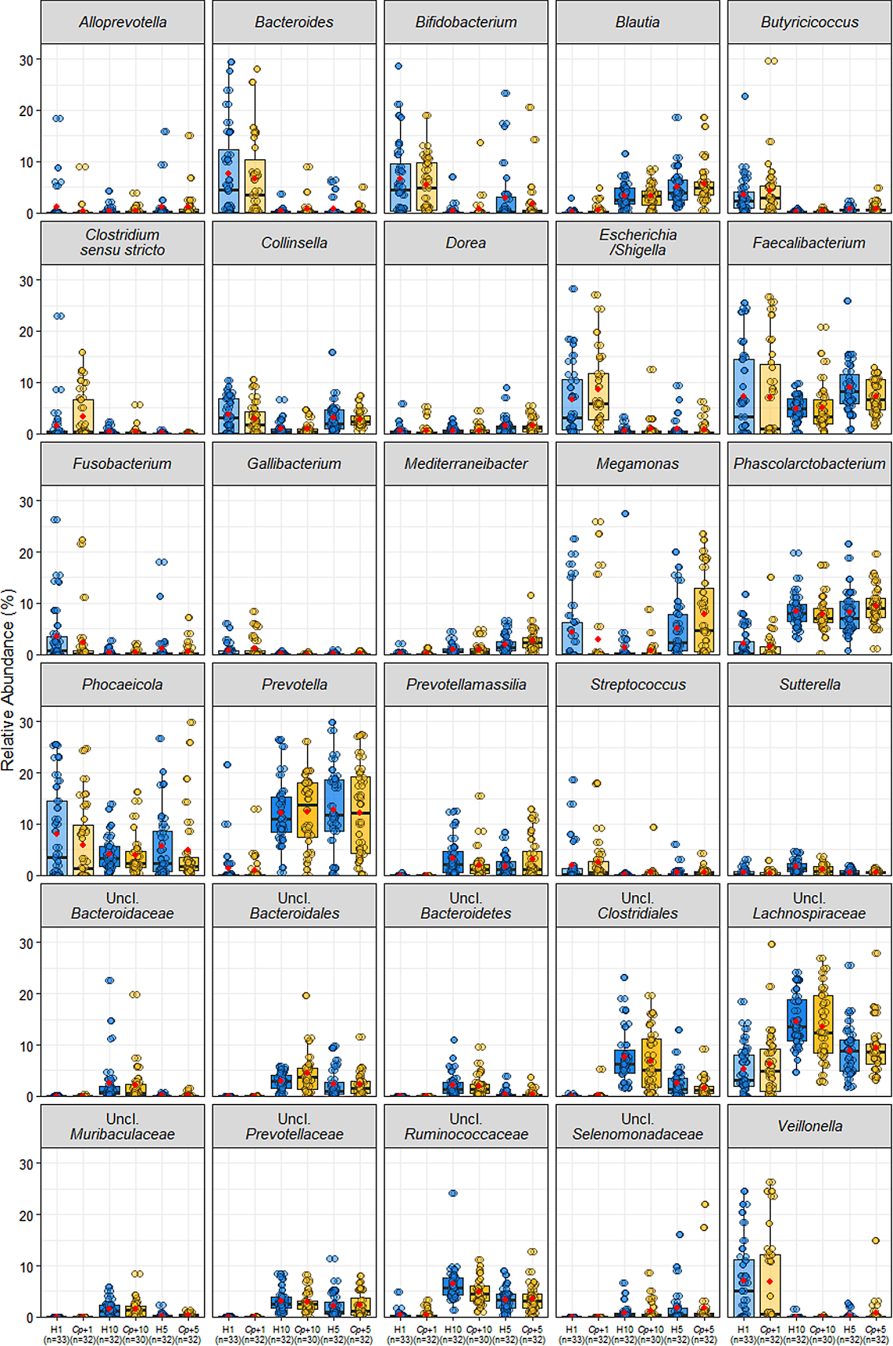
The most abundant genera experienced changes in relative abundance over time. Relative abundance of the top 30 most abundant genera for healthy control and *Cryptosporidium-*positive groups at Week 1, 5, and 10. The red dot shows the mean relative abundance

At the genus level, the relative abundances of *Escherichia*/*Shigella* (*q* = 2.75E-09), *Veillonella* (*q* = 2.75E-09), *Bacteroides* (*q* = 6.93E-09), *Clostridium sensu stricto* (*q* = 1.79E-04), *Megamonas* (*q* = 1.35E-01), *Collinsella* (*q* = 4.36E-06), *Bifidobacterium* (*q* = 8.72E-07) significantly decreased over the three time-points (Figs. 3B and 4). Genera that significantly increased over the three time-points included *Phascolarctobacterium* (*q* = 3.91E-14), *Prevotella* (*q* = 3.43E-12), *Blautia (q* = 4.99E-05), and unclassified genera of the *Lachnospiraceae* family (*q* = 4.57E-14), *Ruminococcaceae* family (*q* = 6.92E-26), and *Clostridiales* order (*q* = 5.90E-21; Figs. 3B and 4; Additional file 11). However, there were no significantly different genera between H and *Cp*+ groups within any of the time-points (Additional file 12). When analysed separately and together neither Farm 2 or Farm 3 showed any significant differences in genera composition between the H and *Cp*+ groups within each time-point (Additional file 13–18).

The results indicated that there were no statistically significant differences in any genera between H and *Cp*+ groups within W1, W5 and W10 time-points (*q* ≥ 0.25; Additional file 12). This would suggest that the pre-scour relative abundance of genera in the faecal microbiome does not predispose calves to *C. parvum* infection and that *C. parvum* infection does not significantly impact the long-term development of the faecal microbiome post-infection in a farm setting.

A core microbiome was established for H and *Cp*+ groups at each time-point with an abundance of ≥ 0.01% in ≥ 80% of the samples (Fig. 3C, Additional file 19). H and *Cp*+ calves shared the majority of their genera but had some taxa that were found exclusively within each group at the stated abundance and prevalence (Fig. 3C, Additional file 19). The genus found exclusively in the H1 group was an unclassified genus of the *Enterococcaceae* family, whilst *Anaerobutyricum*, *Holdemanella*, *Succiniclasticum*, *Escherichia/Shigella*, and *Gallibacterium* were the genera found in H5, and *Ihubacter*, *Anaerobutyricum*, *Dorea*, and *Roseburia* in the H10. The genera found exclusively in *Cp*+1 group were *Enterococcus* and an unclassified genus of the *Lactobacillales* order, then *Alloprevotella*, *Limosilactobacillus*, an unclassified genus of the *Clostridiales incertae sedis* XIII family, *Ruminococcus* 2, and an unclassified genus of the *Erysipelotrichaceae* family were found in *Cp*+5, and there were no taxa exclusive to *Cp*+10. The unique taxa detected in each group were present at extremely low abundance (< 1%) and we do not yet understand the impact of rare taxa on host health or in the context of disease.

### Antibiotic and anti-cryptosporidial treatment impacts the relative abundance of some taxa in the calf faecal microbiome

Antibiotic treatment that was administered on Farm 2 influenced the relative abundance of certain taxa in the microbiome, irrespective of *C. parvum* infection. In the overall analysis, Trimethoprim/sulfadiazine was associated with a statistically significant reduction in *Enterococcus* (*q* = 4.99E-02), *Lactococcus* (*q* = 1.65E-01), and an unclassified genus of the *Atopobiaceae* family (*q* = 2.27E-01) in W5 samples, and a statistically significant reduction in Peptacetobacter (*q* = 1.65E-01) in W10 samples (Additional file 12). The antibiotic, amoxicillin/clavulanic acid was also prescribed to some calves and significantly increased the abundance of *Bacteroides* (*q* = 1.29E-01) and *Paraeggerthella* (*q* = 1.29E-01) within the first week of life (Additional file 12). Tulathromycin was only administered after W1 sampling, and it significantly reduced the relative abundance of *Agathobaculum* (*q* = 2.40E-01) in the W5 samples (Additional file 12). In W10 samples, tulathromycin raised the relative abundance of an unclassified genus of the *Clostridia* class (*q* = 1.65E-01), whilst a reduction in the relative abundance of *Coprococcus* (*q* = 1.65E-01) was observed (Additional file 12). Halofuginone lactate was prescribed either prophylactically or therapeutically and was significantly associated with an increase in the relative abundance of *Porphyromonas* (*q* = 1.06E-01) in W5 samples (Additional file 12). When the samples were grouped separately by farm to determine the effect of the antibiotics on Farm 2, only amoxicillin/clavulanic acid continued to obtain a significantly higher abundance of *Bacteroides* (*q* = 2.16E-01) and *Paraeggerthella* (*q* = 1.79E-01) within the first week of life on Farm 2 (Additional file 16). None of the other antibiotic treatment reached significance in the Farm 2 pairwise comparison. The reanalysis without Farm 1 (Farm 2 and 3) showed there were no significant differences in composition between Farm 2 and 3 when Farm 1 was excluded, though the majority of antibiotic and halofuginone related compositional differences were still significant (Additional file 14). Though the taxa associated with treatment may be present at a low abundance in the microbiome, a change in their proportion due to antibiotic usage could still impact overall calf health and recovery from infection.

### *Cryptosporidium parvum* infection may influence body condition, but does not significantly impact long-term calf weight gain or respiratory health

**Fig. 5:**
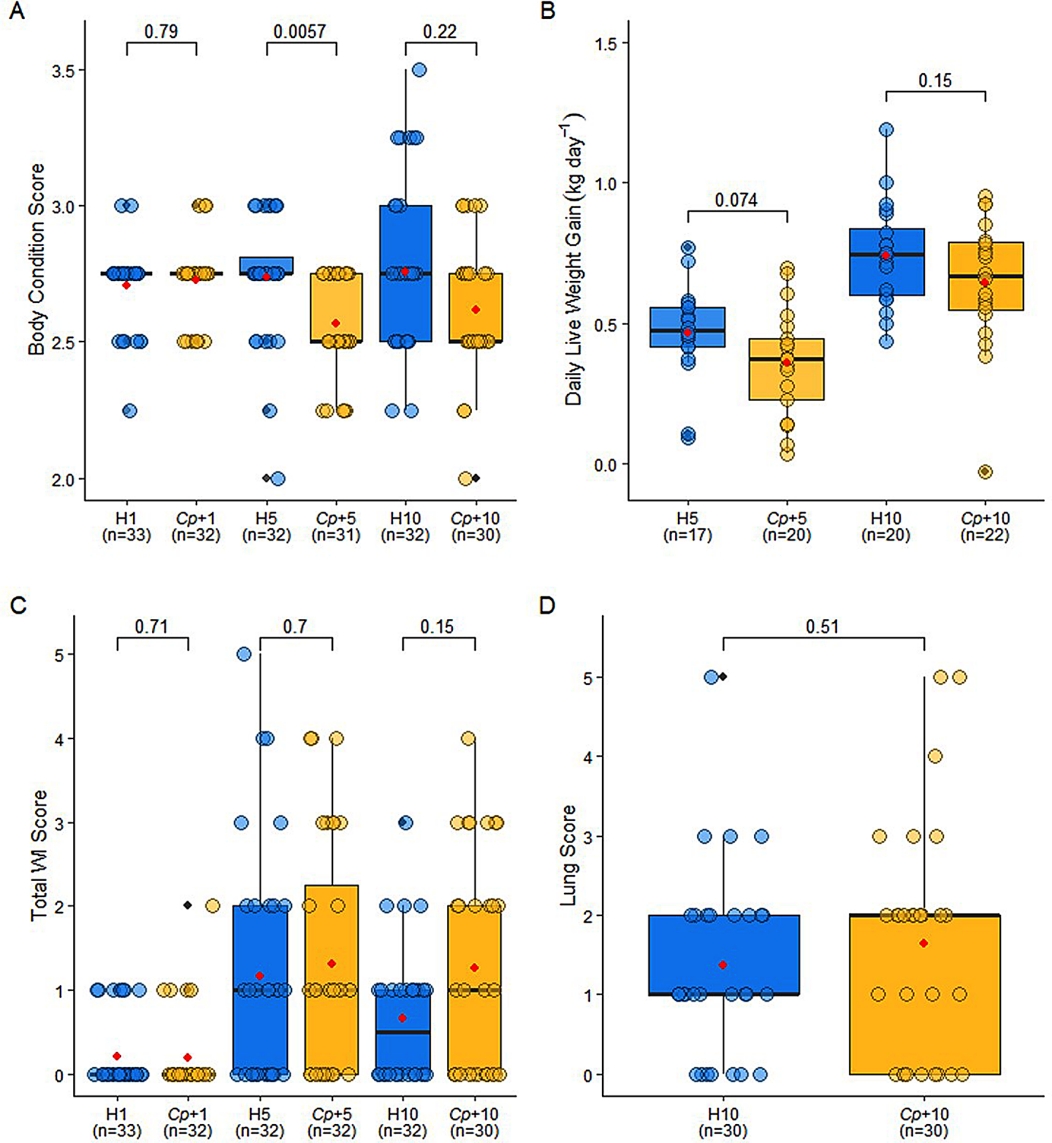
Body condition scores were impacted by past *Cryptosporidium parvum* infection, but daily live weight gain and respiratory disease scoring was not associated with *Cryptosporidium parvum* infection. **A** Body condition scores of healthy control and *Cryptosporidium-*positive groups at Week 1, 5, and 10. **B** Daily live weight gain in kilograms per day of healthy control and *Cryptosporidium*-positive groups at Week 5 and 10 on Farm 2. **C** Total Wisconsin scores for healthy control and *Cryptosporidium-*positive groups at Week 1, 5, and 10. **D** Lung scores for healthy control and *Cryptosporidium-*positive groups at Week 10. The red dots indicate the mean

As *C. parvum* infection did not significantly impact upon the development of the microbiome, and the composition of the microbiome has previously been linked to growth, we wanted to determine if the results of this analysis were reflected in other indicators of calf health including body condition score (BCS), daily live weight gain (DLWG; kg day^− 1^), total Wisconsin (WI) scores and lung scores. When compared at each time-point, BCS was significantly higher in the healthy control group compared to the *Cryptosporidium*-positive group at W5 (Fig. 5A, H5 vs. *Cp*+5; *p* = 0.0057). However, this difference was no longer significant by W10 (Fig. 5A, H10 vs. *Cp*+10; *p* = 0.22). As part of the health monitoring that was conducted throughout the study, calves on Farm 2 were weighed at each time-point. The data showed that the DLWG was not significantly impacted by *C. parvum* infection (Fig. 5B, H5 vs. *Cp*+5; *p* = 0.074, H10 vs. *Cp*+10; *p* = 0.15). This would suggest that the infected dairy calves appeared to have sufficient resilience to maintain an adequate BCS and DLWG post-infection.

Calves also received a WI score (0–3) for four signs of respiratory disease including a cough, nasal discharge, eye, and ear score. The sum of these scores reflects the severity of respiratory disease. Calves with two or more clinical parameters scoring 2–3 or a total score of ≥ 5 are considered to have respiratory disease. In W10, calves underwent thoracic ultrasonography to detect historic lung damage and were given a lung score (0–5). Comparison of total WI scores and lung scores between H and *Cp*+ groups showed that there was no statistically significant difference between the groups (Fig. 5C-D). In spite of this, *Cp*+10 calves had a tendency towards higher WI scores than H10 calves (Fig. 5C; *p* = 0.15), suggesting that *C. parvum* may have some influence on the severity of respiratory infection, which could be attributed to the weakening of the immune system as a result of infection. However, further research would be required to confirm this hypothesis.

## Discussion

The main aim of this study was to describe differences between the faecal microbiomes of pre- and post-infection calves that developed cryptosporidiosis or remained healthy, to determine if *C. parvum* impacts the overall progression of the microbiome during the preweaning stage of life. Based on previous studies that investigated the calf microbiome during active *C. parvum* infection, we predicted that there would be significant differences in certain bacterial taxa in the *Cp*+ group compared to the H group prior to and following infection [7, 18, 19, 38, 39]. Despite some studies showing statistically significant taxa differences between the microbiomes of diarrhoeic *Cp*+ calves and the microbiomes of healthy control calves, it appears that the pre-infection faecal microbiome does not show any features that may predispose calves to cryptosporidiosis [7, 18–20]. This result is in line with the previously cited study that we conducted which examined the microbiome of the same calves prior to *C. parvum* infection using shotgun sequencing, and found no statistically significant differences in diversity or composition between H and *Cp*+ groups in the W1 time-point [21]. A recently published study that investigated the role of the calf faecal microbiome before, during, and after *Cryptosporidium* infection in the first three weeks of life, was unable to determine if any specific taxa were associated with a predisposition to *C. parvum* infection, though significant differences in composition and diversity were observed during diarrhoeal disease [20]. While the relative abundance of the phylum, *Firmicutes*, was found to be significantly higher in the *Cp*+5 group compared to the H5 group, this difference did not remain significant in the W10 time-point, suggesting that *C. parvum* infection may not have a long-lasting impact on the faecal microbiome. This increased abundance of *Firmicutes* was not reflected in a specific genus, suggesting that the combined increase of multiple genera of the phylum *Firmicutes* are responsible for the significant difference observed at the phylum level. A higher abundance of *Firmicutes* has previously been observed in *C. parvum* infected mice, however another study reported a lower relative abundance of *Firmicutes in C. parvum* infected mice compared to uninfected controls, which only emphasises the considerable variability between the microbiomes of *C. parvum* infected hosts [38, 39]. Interestingly, *Faecalibacterium* was present at a higher median relative abundance in the H group compared to the *Cp*+ group at every time-point. Whilst this difference was not statistically significant, another study has shown a positive association between *Faecalibacterium* and better health, improved growth, and reduced incidence of diarrhoea in dairy calves, implying the probiotic potential of this bacterium [40]. Other studies have observed a specific increase in *Fusobacterium* during infection, however, there was no statistically significant difference in this bacterium between H and *Cp*+ groups before or after infection in this study [18–20]. In addition, the study outcome indicates that in a farm setting, there is no particular microbial composition or shared pre-disposing factor that could be targeted therapeutically/prophylactically. This is noteworthy, as in an experimental setting, certain taxa may be shown to predispose animals to infection, yet targeting these taxa in a farm setting may not be beneficial for the majority of animals. The results of this study also imply that the dysbiosis observed in the faecal microbiome of calves with an active infection in other studies may be caused by direct parasite interactions with the microbiota. However, further research is necessary to confirm this hypothesis. The outcome of our study paints a positive picture of the impact of *C. parvum* infection on the microbiome in dairy calves, as there was no evidence that bovine cryptosporidiosis led to long-term detrimental effects on the development of the calf microbiome, which is essential for good health and the development of a competent immune system.

Whilst the impact of *C. parvum* on the pre- and post-infection calf faecal microbiome was minimal, the changes in diversity and the relative abundance of several taxa over time were substantial. There are various studies that have examined the development of the calf faecal microbiome, particularly during the first week of life, as this is the critical stage when calves are most vulnerable to infection [9, 15, 40–46]. The majority of studies correspond with our results showing an increase in alpha diversity and a reduction in Bray-Curtis beta diversity with age, and that the most abundant phyla prior to weaning are *Firmicutes*, *Proteobacteria*, *Bacteroidetes*, and *Actinobacteria*. Several studies with comparable results showed that during the preweaning period, *Faecalibacterium* relative abundance increased and then declined again, *Bacteroides* and *Escherichia*/*Shigella* are highly abundant in the first week of life and then drop off over time, whilst *Ruminococceae*, *Lachnospiraceae,* and *Prevotella* became more abundant later in the pre-weaning period [9–11, 47–49]. Other studies also exhibited trends not observed in our data, for example Uyeno et al. (2010) showed relatively high levels of *Bacteroides*-*Prevotella* (33.0-60.6%) and *Clostridium*-*Eubacterium* (9.90–19.0%) groups between 1 and 12 weeks of age [46]. However, in our study, these genera made up a much smaller proportion of the overall composition and were far more varied in abundance between time-points. There is a lot of variation in the taxa that may characterise different age groups between calf faecal microbiome studies largely due to differences in study design, sample and data processing, diet, breed, location, and farm management practices. The variation during the preweaning stage may create a challenge in the development of efficacious pre-/pro-/post-biotics for the maintenance of healthy calves or to mitigate disease.

The administration of various antibiotics had an effect on the relative abundance of certain taxa in the faecal microbiome of the calves in the overall analysis. This was an expected outcome as it is well-documented that antibiotics can alter the calf gut microbiome [50–60]. Most of these studies did not use the particular antibiotics used on Farm 2 in this study, and often studied the effect of a mixture of antibiotics or waste milk containing antibiotic residues. Whilst the majority of these studies found significant differences in the composition and diversity of treated and untreated calves, two studies found minimal differences caused by antibiotic treatment [54, 60]. This shows that changes in the diversity and composition of the faecal microbiome may be dependent on the type of antibiotic, the dosage, the duration of exposure, and its mode of action. When Farm 2 was separated out as the only farm where calves received antibiotic treatment, amoxicillin and clavulanic acid maintained significant differences in the taxa in W1 samples comparable with the overall analysis. Despite this, compositional differences observed in the overall analysis for trimethoprim/sulfadiazine did not reach significance in the separate Farm 2 analysis. This would suggest that while the administration of antibiotics may have some effect on the composition of the microbiome, treatment did not appear to impact the susceptibility to or recovery from *C. parvum* infection in this study. In the overall analysis of our study, tulathromycin seemed to have the most impact on taxa in the order *Clostridiales*, having caused a statistically significant change in the relative abundance of genera within this taxon. A study looking at the impact of antibiotics on the faecal microbiome also found that treatment had the most influence on the order of *Clostridiales* [61]. Martin et al. showed that tulathromycin reduced the relative abundance of *Bifidobacterium* within the first week of life and increased the abundance of *Escherichia*/*Shigella* at approximately 3 weeks of age [62]. In two other studies that investigated the effect of tulathromycin on the faecal microbiome in cattle, they found that this antibiotic had no statistically significant impact on the microbiome [63, 64]. Differences in the study design, sample and data processing, diet, breed, location, and farm management practices likely account for the inconsistencies observed between studies.

The only step farmers took to mitigate cryptosporidiosis on the participating farms was the administration of halofuginone lactate (100 µg/ kg (body weight), once a day for 7 consecutive days) either prophylactically (Farm 1 and 3) or therapeutically (Farm 2), though this was not always consistent. Therefore, a starker difference between healthy and infected calf microbiomes may have been observed pre- and post-infection had the calves not received anti-cryptosporidial treatment. We found that halofuginone lactate had a positive association with *Porphyromonas* in the overall analysis. Porphyromonadaceae bacterium DJF B175 has been associated with high daily weight gain in calves, so perhaps halofuginone lactate could improve weight gain in calves if it also promotes this type of bacteria [11]. Dorbek-Kolin et al. shows that calves that received halofuginone lactate had a higher mean relative abundance of *Actinobacteria* and higher microbial diversity during a cryptosporidiosis outbreak compared to untreated calves [18]. While this trend was not observed in our study, the current literature is not sufficient to explain the associations that were identified. When Farm 2 was analysed alone, halofuginone lactate did not significantly impact the composition of the faecal microbiome, though this could be due to a smaller sample size, and inconsistent dosing across the diarrhoeic calves.

Our findings may suggest that *C. parvum* infection does not significantly affect daily live weight gain in dairy calves, though BCS may be affected in the short-term post-infection. The significantly elevated BCS of H5 calves compared to *Cp*+5 calves were likely due to some weight loss that occurred as a result of the diarrhoea in the calves that developed *C. parvum* infection. Though DLWG did not appear to be significantly impacted by prior infection, it could be attributed to the treatment of *Cp*+ calves with halofuginone lactate following the diagnosis of cryptosporidiosis on Farm 2, reducing the severity of infection, and leading to the mitigation of weight loss. Niine et al. showed that correct treatment with halofuginone lactate given to calves with cryptosporidiosis was negatively associated with weight gain compared to untreated infected calves at 3 months of age which the authors speculate may be due to the delay and potential expansion of the lifecycle of *C. parvum* induced by halofuginone lactate [65]. In contrast, Shaw et al. showed a statistically significant difference in weight gain at 6 months between healthy calves and calves that developed cryptosporidiosis and were treated with halofuginone lactate [66]. This would suggest that cryptosporidiosis has an impact on long-term weight gain, though this may be dependent on disease severity. To caveat this, investigating the effect of cryptosporidiosis on the DLWG of calves was not the main aim of this study, and therefore the body weight data may be underpowered. Despite this, a numeric difference was observed, and the *p*-values were close to significance, suggesting that a larger sample population and fewer variables would potentially allow the difference to reach significance.

A previous study has shown an association between diarrhoea incidence and respiratory disease, possibly linked to a weakened immune system or inconsistent colostrum supplementation [67]. Another study has shown a specific association between *C. parvum* infection and increased risk of respiratory disease in dairy calves [68]. Though there were some small differences in total WI scores and lung scores between the H and *Cp*+ groups, and the *Cp*+10 calves had a tendency towards higher WI scores than the H10 calves, these differences did not reach significance. Moreover, the administration of metaphylactic antibiotics may have contributed to the overall low severity of respiratory signs observed in the calves. In addition, the healthy control group on Farm 2 was selected for comparable history of antibiotic usage to *Cryptosporidium*-positive group rather than representing all calves that did not develop *C. parvum* infection. Whilst the study was designed to prioritise the collection of faecal samples for microbiome analysis, the recording of respiratory scores was not our primary objective, and therefore the sample size may not provide enough power to detect significant differences.

### Limitations

The study had limitations that may have had an impact on the final results and therefore, we will reflect on them here.

The main limitation of the study design was the use of LFT to diagnose the calves with cryptosporidiosis. Whilst this method of detection has the capacity to detect false positive and negative results, with a sensitivity and specificity of 94.2% and 88.2%, respectively [69, 70], it does have the advantage of being easy-to-use, cost-effective, and fast, especially for a study where great numbers of large animals were required to be tested on a farm environment. The positive LFT coupled with the confirmation of diarrhoea by experienced veterinary clinicians was regarded as adequate criteria for the diagnosis of cryptosporidiosis.

It is challenging to draw robust conclusions on the effect of specific antibiotics from this data as they were only prescribed on one of the farms and therefore significant differences could also be attributed to the farm location or other environmental factors. In addition, all of the taxa were present at an abundance of less than 1% and therefore can be classified as rare or low abundance. While the antibiotics may have affected these taxa, we do not yet understand the role that low abundance taxa play in the microbiome and whether they have a substantial impact on calf health and resistance to infection.

The body weight and respiratory disease data were collected throughout the study as a measure of calf health where possible. Weight was measured using a scale on Farm 2, whilst a weigh tape was used to estimate weight on Farm 1 and 3 at W5 and W10 sampling time-points. Therefore, the data for DLWG may be underpowered as only data from Farm 2 was useable. In addition, the number of calves may be too small to draw any strong conclusions regarding the relationship between cryptosporidiosis and DLWG or respiratory disease over the first 12 weeks of life in this study.

Some samples went missing in transit, which may marginally reduce the power of the study. However, these samples are missing completely at random (MCAR) and therefore do not introduce bias to the study design.

## Conclusion

To summarise, *C. parvum* infection had minimal impact on the microbial diversity and composition of the calf faecal microbiome pre- or post-infection when compared with healthy calves, with age having the greatest impact on the development of the microbiome. This study and our previous shotgun metagenomic work lead us to speculate that the changes observed in other studies may be a result of direct interactions with the parasite during infection and not a consequence of pre-existing microbiome differences. Further research to confirm this theory could include larger sample populations and *Cp*+ calves grouped by pre-diarrhoea, diarrhoea and post-diarrhoea within the same farm environment, without antibiotic use and multiple tests to confirm natural infections. In addition, *C. parvum* does not affect the overall long-term progression of the microbiome on the studied farms. Though this is surprising, it is not an unwelcome outcome as it is indicative of the calf’s ability to restore the gut microbiome post-infection, which is important for overall health and protecting cattle against further gastrointestinal disease.

## Electronic supplementary material

Below is the link to the electronic supplementary material.

Additional file 1: Supplementary Figures

Additional file 2: Analysis Pipeline

Additional file 3: MaAsLin2 Phylum Longitudinal Results

Additional file 4: MaAsLin2 Phylum Pairwise Results

Additional file 5: MaAsLin2 Phylum Farm 2–3 Longitudinal Results

Additional file 6: MaAsLin2 Phylum Farm 2–3 Pairwise Results

Additional file 7: MaAsLin2 Phylum Farm 2 Longitudinal Results

Additional file 8: MaAsLin2 Phylum Farm 2 Pairwise Results

Additional file 9: MaAsLin2 Phylum Farm 3 Longitudinal Results

Additional file 10: MaAsLin2 Phylum Farm 3 Pairwise Results

Additional file 11: MaAsLin2 Genus Longitudinal Results

Additional file 12: MaAsLin2 Genus Pairwise Results

Additional file 13 MaAsLin2 Genus Farm 2–3 Longitudinal Results

Additional file 14: MaAsLin2 Genus Farm 2–3 Pairwise Results

Additional file 15: MaAsLin2 Genus Farm 2 Longitudinal Results

Additional file 16: MaAsLin2 Genus Farm 2 Pairwise Results

Additional file 17: MaAsLin2 Genus Farm 3 Longitudinal Results

Additional file 18: MaAsLin2 Genus Farm 3 Pairwise Results

Additional file 19: Core Microbiome

Additional file 20: Metadata

## Data Availability

Sequence data that support the findings of this study have been deposited in the European Nucleotide Archive under BioProject: PRJEB70717. Data analyses and results are provided within the manuscript or additional files.

## Abbreviations

*C. parvum*: *Cryptosporidium parvum*
*Cp*+: *Cryptosporidium*-positive group
H: Healthy control group
GIT: Gastrointestinal tract
FMT: Faecal microbiota transplantation
LFT: Lateral flow testing
LOESS: Locally estimated scatterplot smoothing
BCS: Body condition score
WI: Wisconsin
CGR: Centre for Genomic Research, University of Liverpool
DLWG: Daily live weight gain
OTU: Operational taxonomic unit
W1: Week 1
W5: Week 5
W10: Week 10
