# Supplementary material for: Progression of the faecal microbiome in preweaning dairy calves that develop cryptosporidiosis
Source: Anim Microbiome. 2025 Jan 6;7:3. doi: 10.1186/s42523-024-00352-1 (PMC11706078; doi:10.1186/s42523-024-00352-1)
Supplement: Supplementary file 19 — Additional file 19: Core Microbiome [file 42523_2024_352_MOESM19_ESM.pptx]

## Slide 1
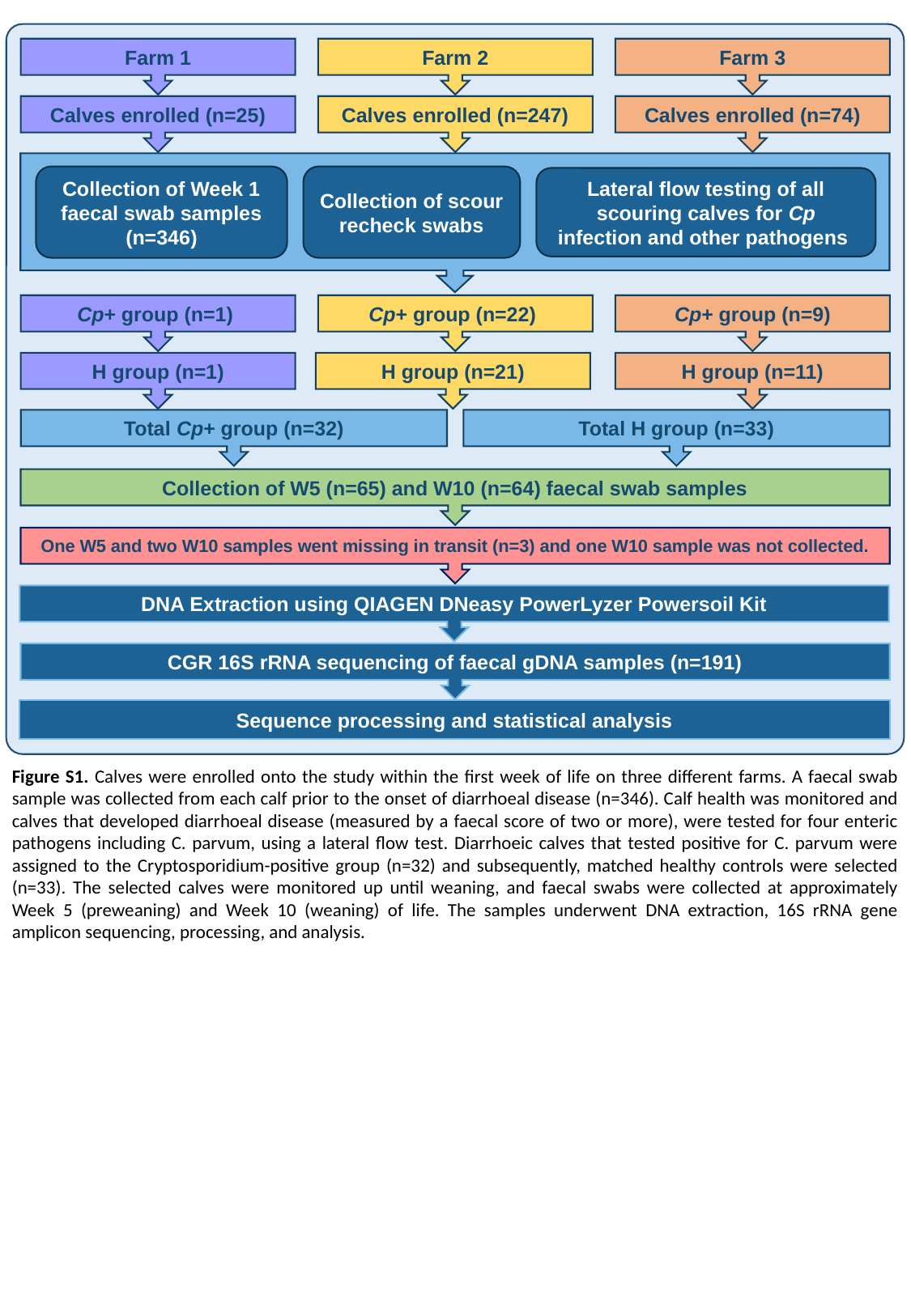

Farm 1
Farm 2
Farm 3
Calves enrolled (n=25)
Calves enrolled (n=247)
Calves enrolled (n=74)
Collection of Week 1 faecal swab samples (n=346)
Collection of scour recheck swabs
Lateral flow testing of all scouring calves for Cp infection and other pathogens
Cp+ group (n=1)
Cp+ group (n=22)
Cp+ group (n=9)
H group (n=1)
H group (n=21)
H group (n=11)
Total Cp+ group (n=32)
Total H group (n=33)
Collection of W5 (n=65) and W10 (n=64) faecal swab samples
One W5 and two W10 samples went missing in transit (n=3) and one W10 sample was not collected.
DNA Extraction using QIAGEN DNeasy PowerLyzer Powersoil Kit
CGR 16S rRNA sequencing of faecal gDNA samples (n=191)
Sequence processing and statistical analysis
Figure S1. Calves were enrolled onto the study within the first week of life on three different farms. A faecal swab sample was collected from each calf prior to the onset of diarrhoeal disease (n=346). Calf health was monitored and calves that developed diarrhoeal disease (measured by a faecal score of two or more), were tested for four enteric pathogens including C. parvum, using a lateral flow test. Diarrhoeic calves that tested positive for C. parvum were assigned to the Cryptosporidium-positive group (n=32) and subsequently, matched healthy controls were selected (n=33). The selected calves were monitored up until weaning, and faecal swabs were collected at approximately Week 5 (preweaning) and Week 10 (weaning) of life. The samples underwent DNA extraction, 16S rRNA gene amplicon sequencing, processing, and analysis.

## Slide 2
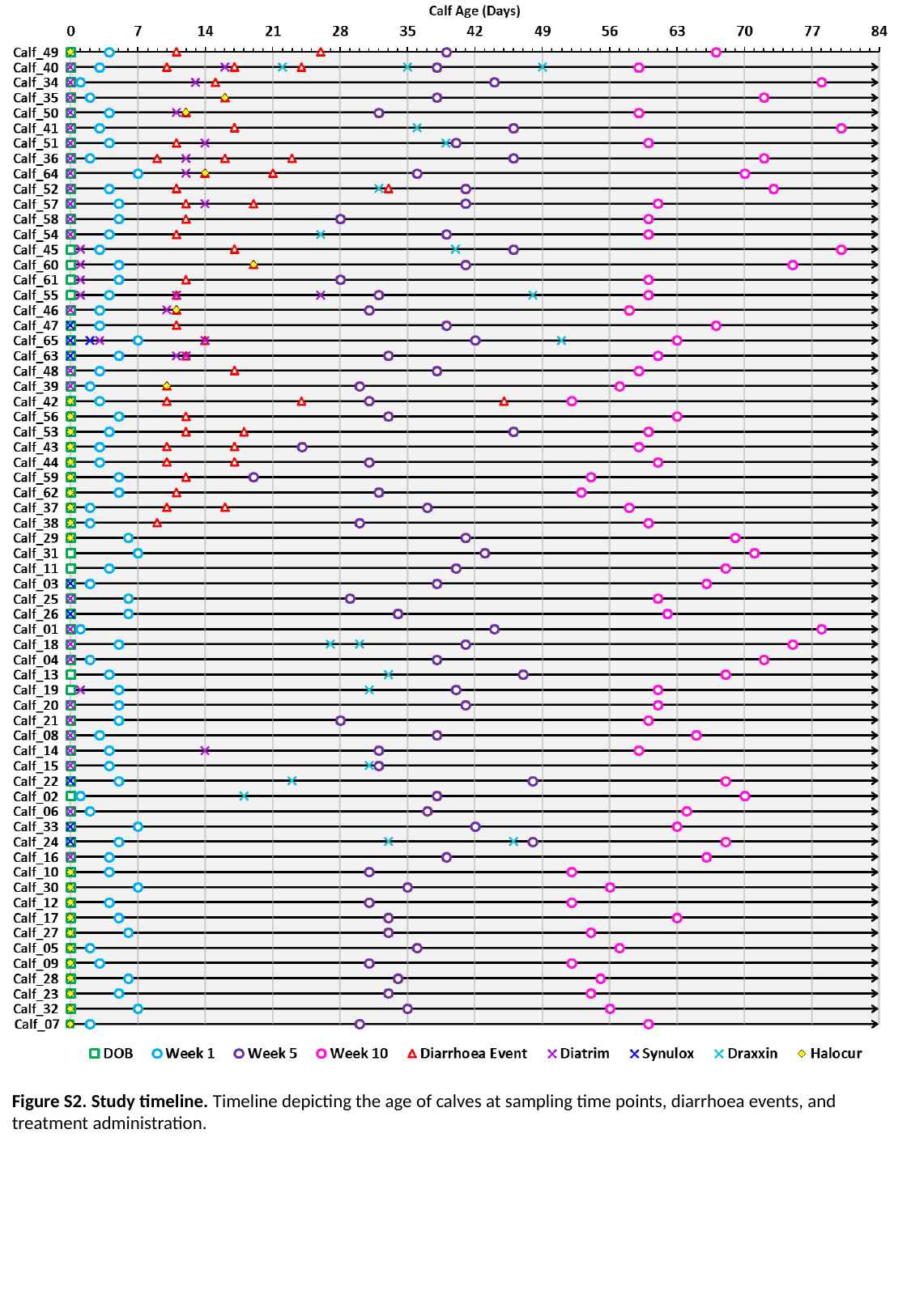

Figure S2. Study timeline. Timeline depicting the age of calves at sampling time points, diarrhoea events, and treatment administration.

## Slide 3
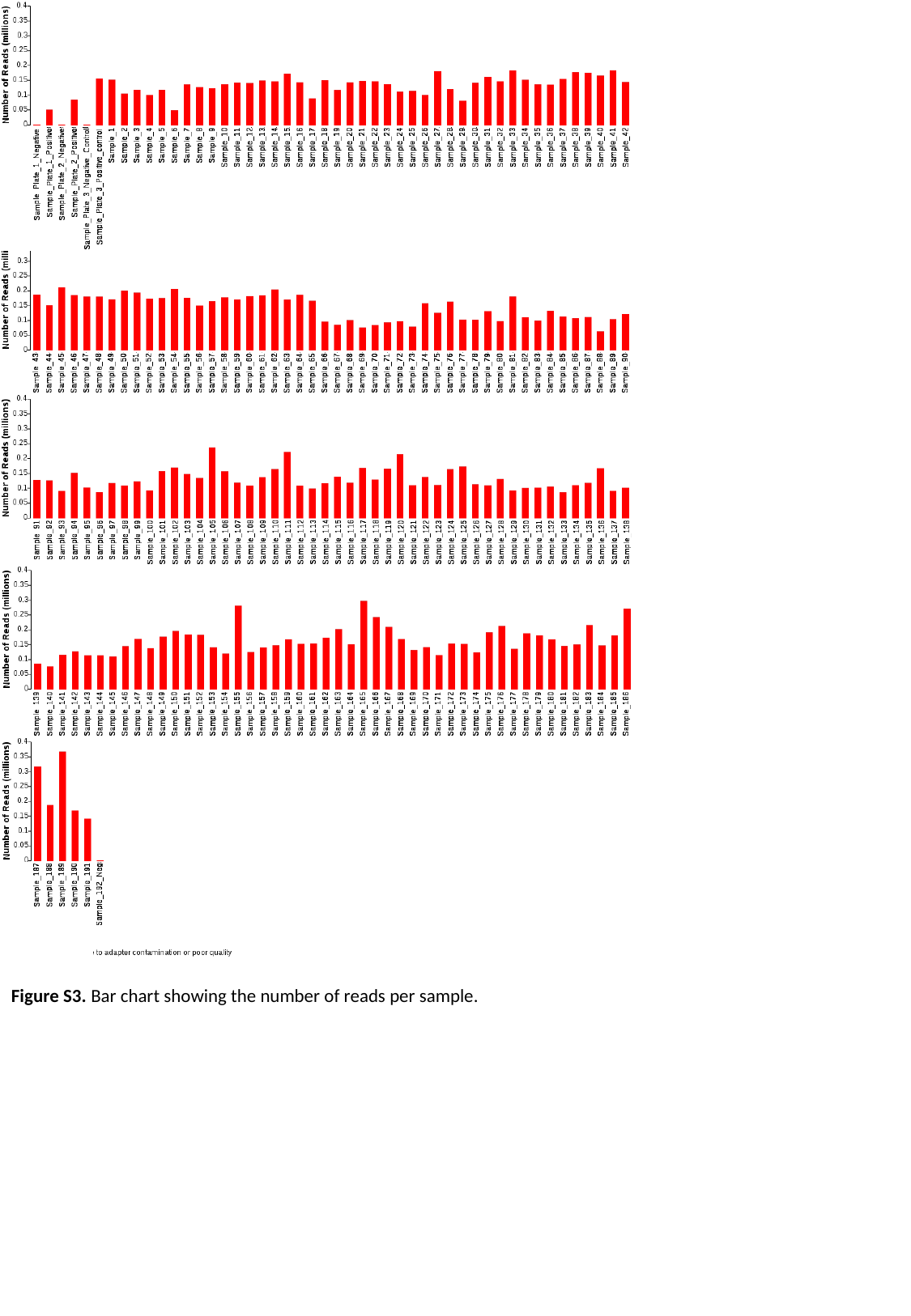

Figure S3. Bar chart showing the number of reads per sample.

## Slide 4
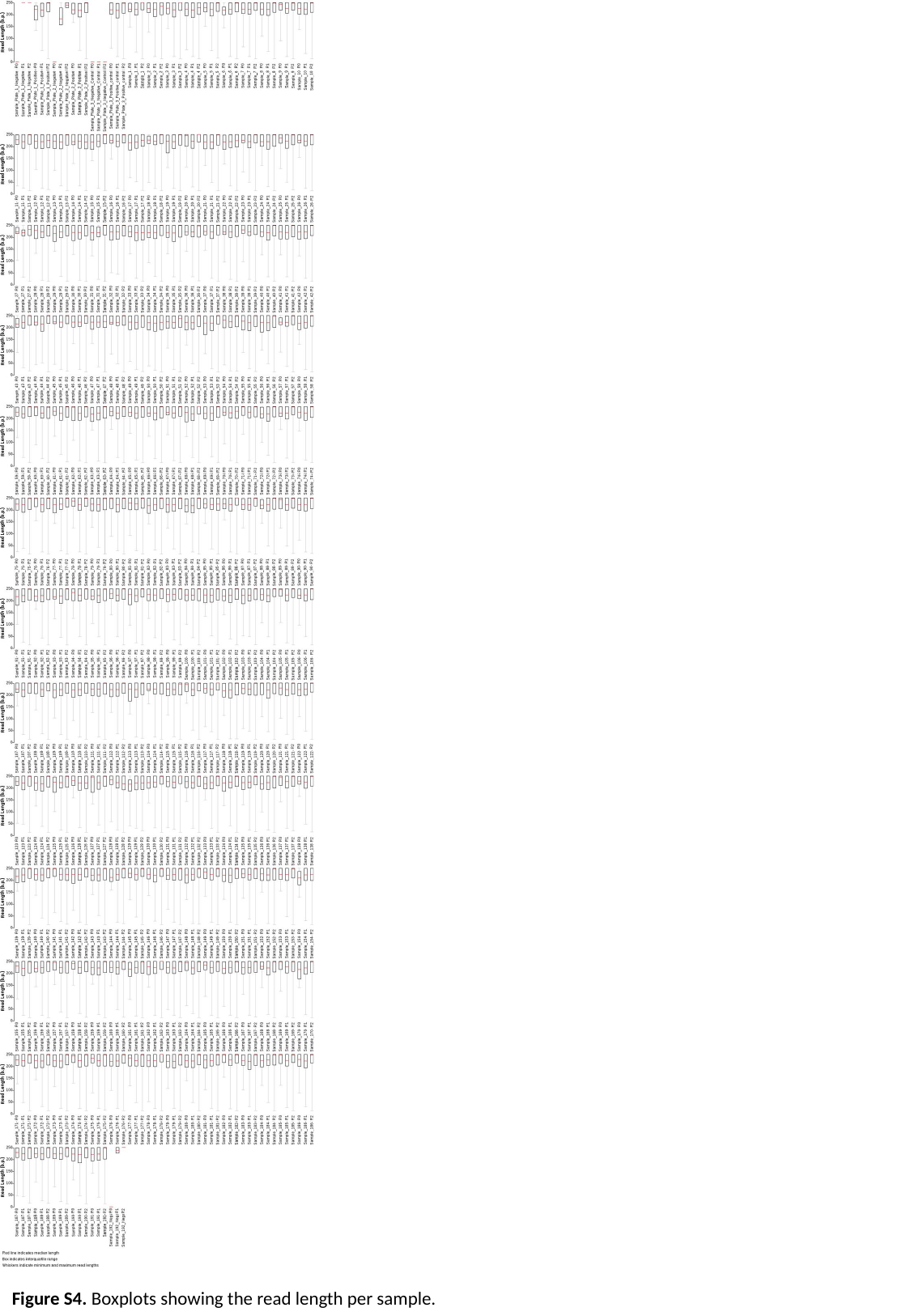

Figure S4. Boxplots showing the read length per sample.

## Slide 5
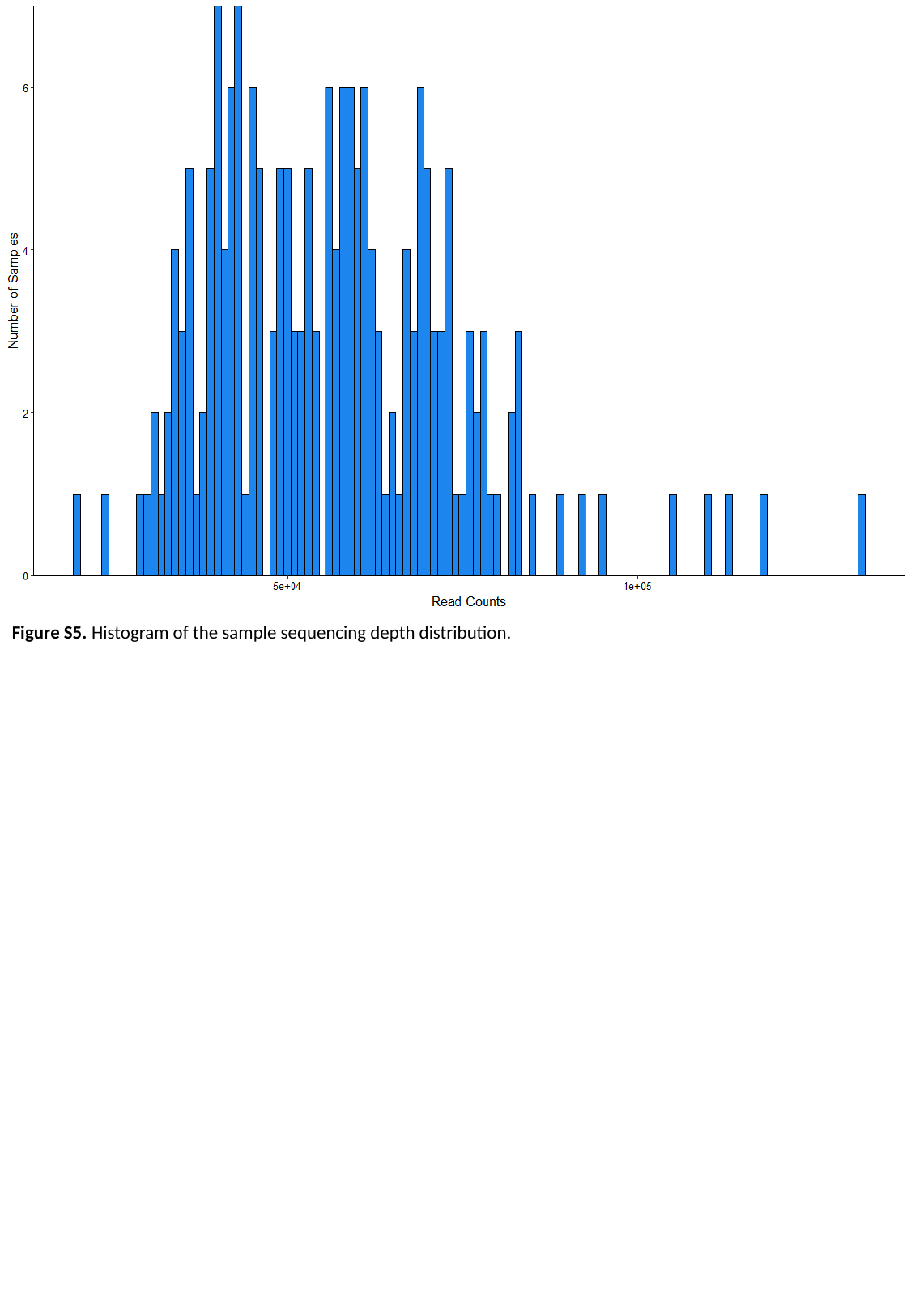

Figure S5. Histogram of the sample sequencing depth distribution.

## Slide 6
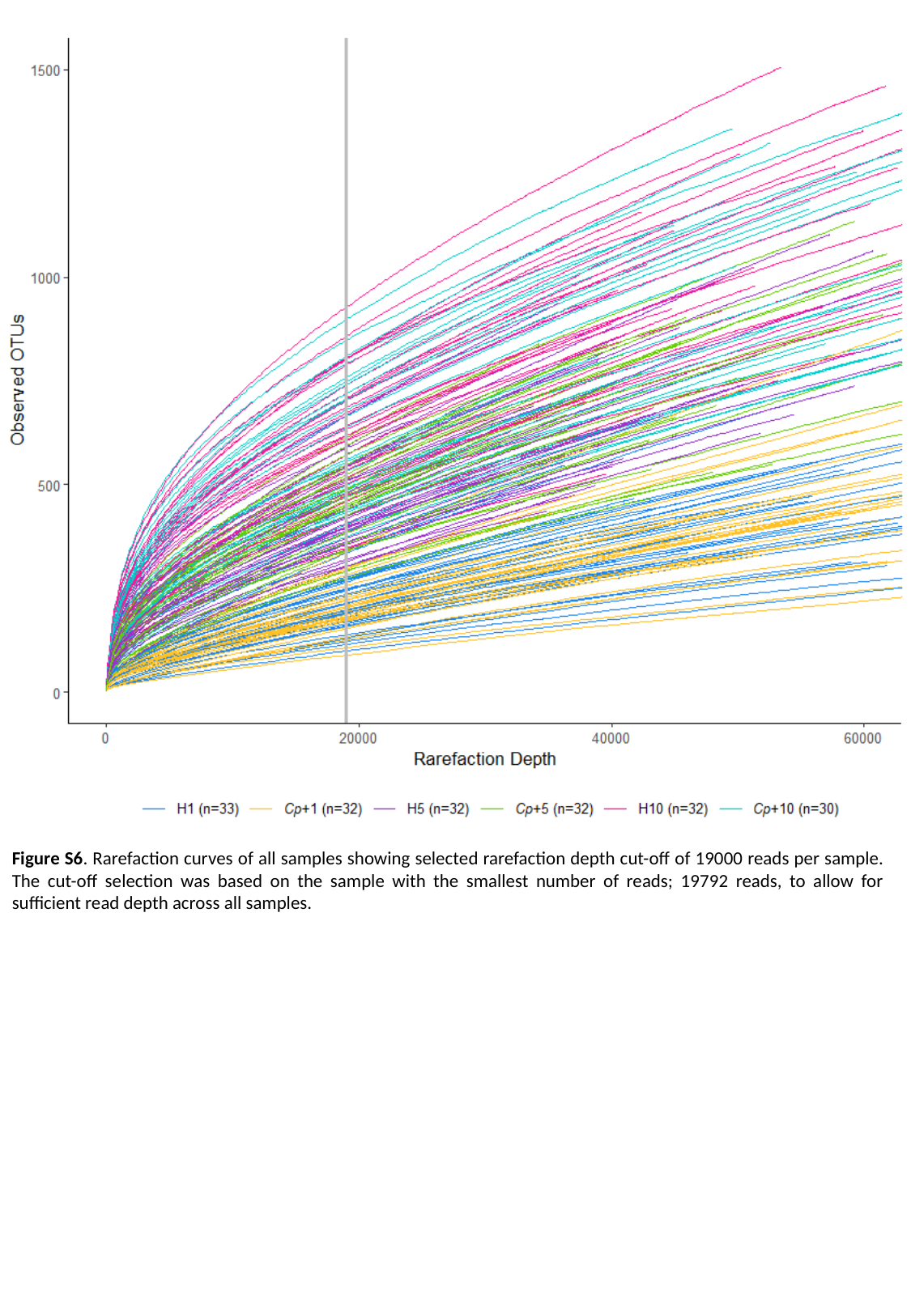

Figure S6. Rarefaction curves of all samples showing selected rarefaction depth cut-off of 19000 reads per sample. The cut-off selection was based on the sample with the smallest number of reads; 19792 reads, to allow for sufficient read depth across all samples.

## Slide 7
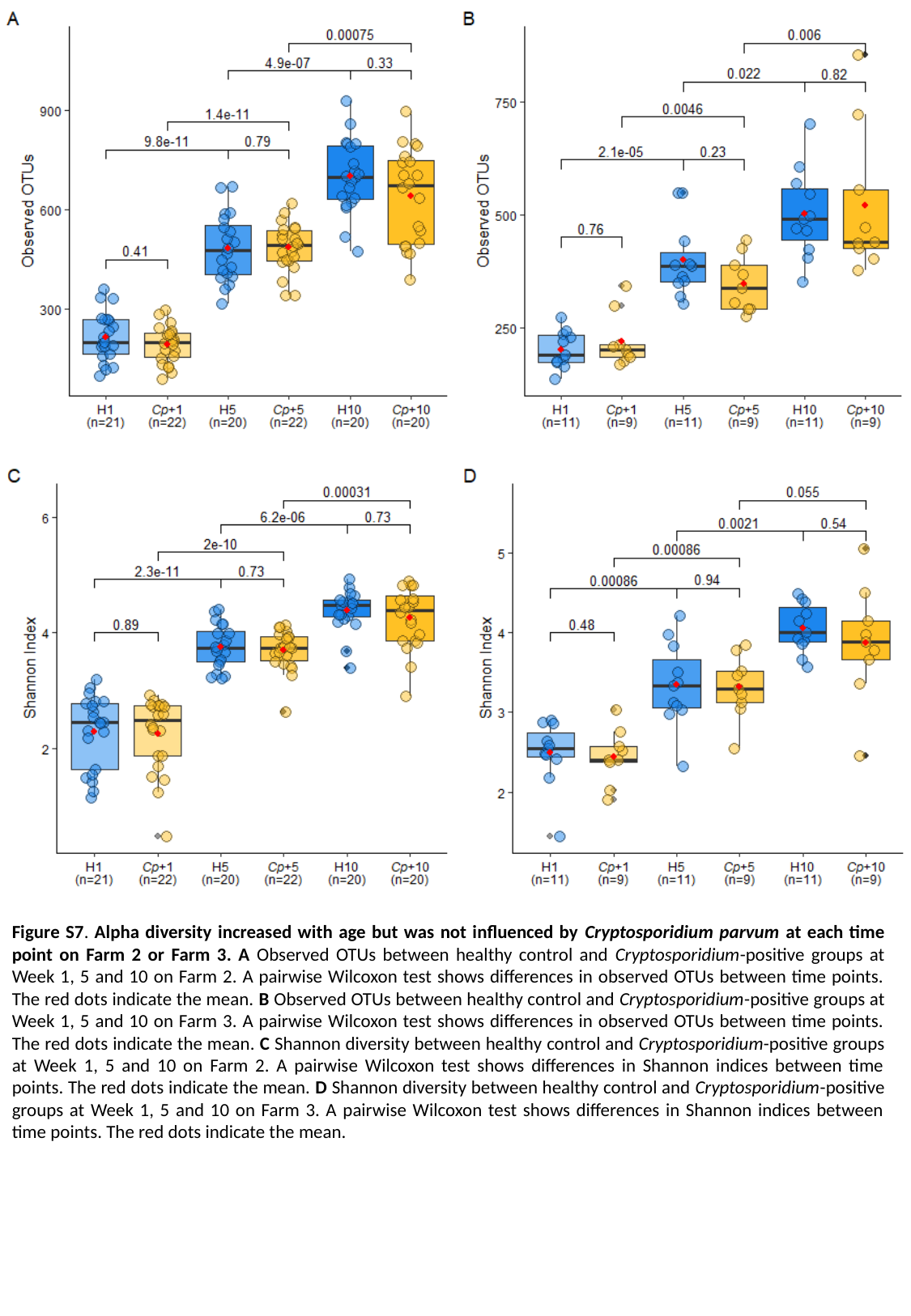

Figure S7. Alpha diversity increased with age but was not influenced by Cryptosporidium parvum at each time point on Farm 2 or Farm 3. A Observed OTUs between healthy control and Cryptosporidium-positive groups at Week 1, 5 and 10 on Farm 2. A pairwise Wilcoxon test shows differences in observed OTUs between time points. The red dots indicate the mean. B Observed OTUs between healthy control and Cryptosporidium-positive groups at Week 1, 5 and 10 on Farm 3. A pairwise Wilcoxon test shows differences in observed OTUs between time points. The red dots indicate the mean. C Shannon diversity between healthy control and Cryptosporidium-positive groups at Week 1, 5 and 10 on Farm 2. A pairwise Wilcoxon test shows differences in Shannon indices between time points. The red dots indicate the mean. D Shannon diversity between healthy control and Cryptosporidium-positive groups at Week 1, 5 and 10 on Farm 3. A pairwise Wilcoxon test shows differences in Shannon indices between time points. The red dots indicate the mean.

## Slide 8
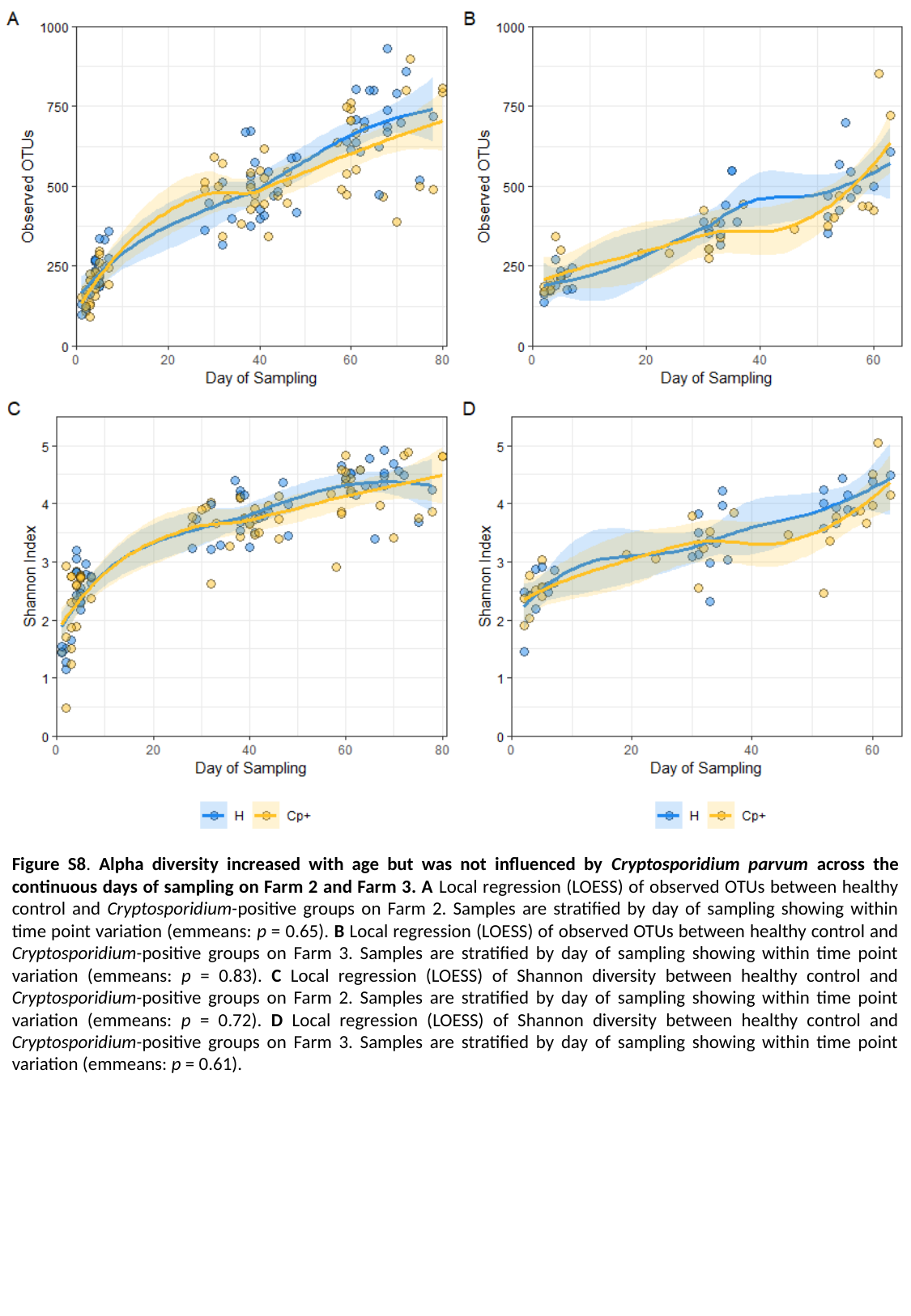

Figure S8. Alpha diversity increased with age but was not influenced by Cryptosporidium parvum across the continuous days of sampling on Farm 2 and Farm 3. A Local regression (LOESS) of observed OTUs between healthy control and Cryptosporidium-positive groups on Farm 2. Samples are stratified by day of sampling showing within time point variation (emmeans: p = 0.65). B Local regression (LOESS) of observed OTUs between healthy control and Cryptosporidium-positive groups on Farm 3. Samples are stratified by day of sampling showing within time point variation (emmeans: p = 0.83). C Local regression (LOESS) of Shannon diversity between healthy control and Cryptosporidium-positive groups on Farm 2. Samples are stratified by day of sampling showing within time point variation (emmeans: p = 0.72). D Local regression (LOESS) of Shannon diversity between healthy control and Cryptosporidium-positive groups on Farm 3. Samples are stratified by day of sampling showing within time point variation (emmeans: p = 0.61).

## Slide 9
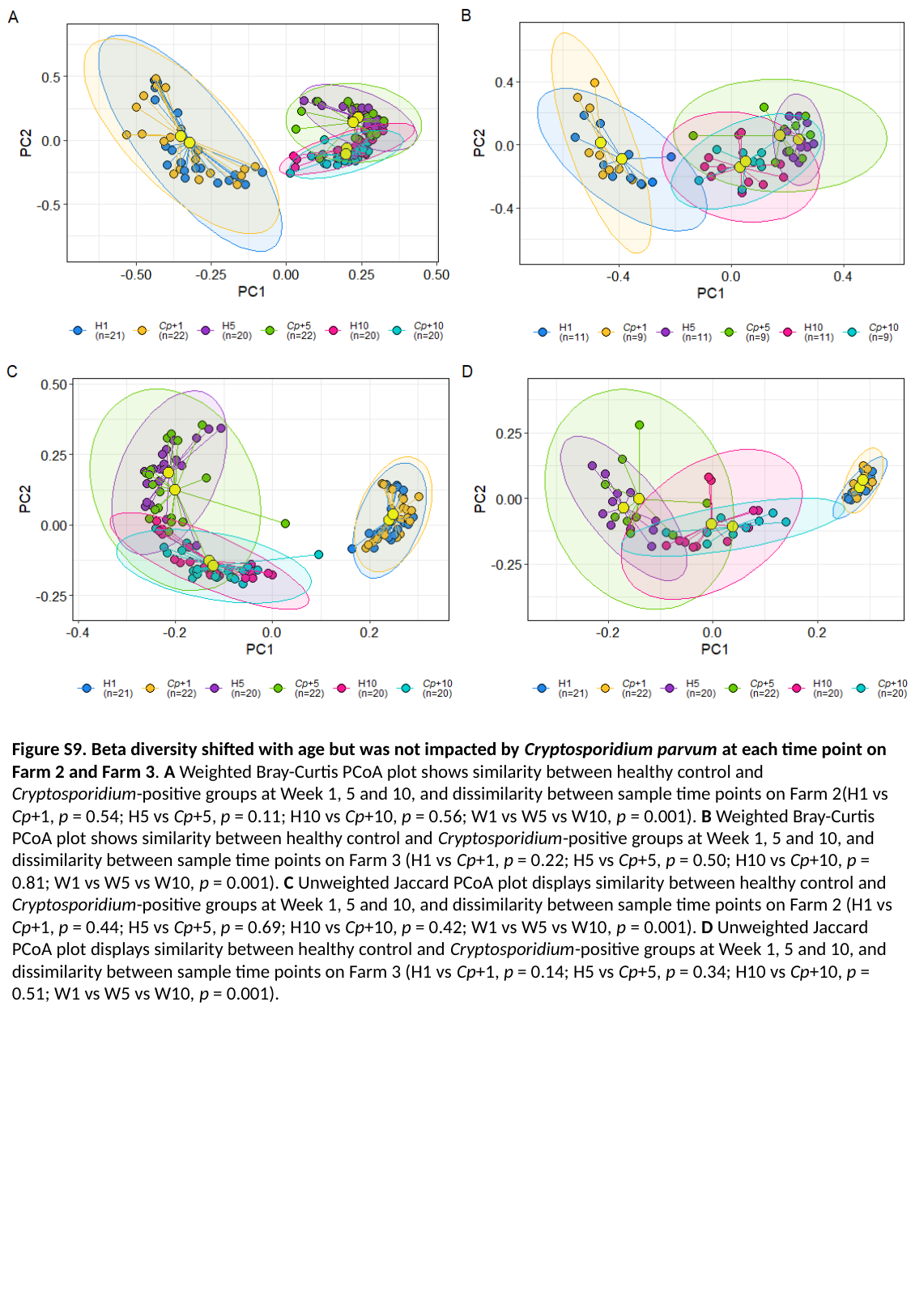

Figure S9. Beta diversity shifted with age but was not impacted by Cryptosporidium parvum at each time point on Farm 2 and Farm 3. A Weighted Bray-Curtis PCoA plot shows similarity between healthy control and Cryptosporidium-positive groups at Week 1, 5 and 10, and dissimilarity between sample time points on Farm 2(H1 vs Cp+1, p = 0.54; H5 vs Cp+5, p = 0.11; H10 vs Cp+10, p = 0.56; W1 vs W5 vs W10, p = 0.001). B Weighted Bray-Curtis PCoA plot shows similarity between healthy control and Cryptosporidium-positive groups at Week 1, 5 and 10, and dissimilarity between sample time points on Farm 3 (H1 vs Cp+1, p = 0.22; H5 vs Cp+5, p = 0.50; H10 vs Cp+10, p = 0.81; W1 vs W5 vs W10, p = 0.001). C Unweighted Jaccard PCoA plot displays similarity between healthy control and Cryptosporidium-positive groups at Week 1, 5 and 10, and dissimilarity between sample time points on Farm 2 (H1 vs Cp+1, p = 0.44; H5 vs Cp+5, p = 0.69; H10 vs Cp+10, p = 0.42; W1 vs W5 vs W10, p = 0.001). D Unweighted Jaccard PCoA plot displays similarity between healthy control and Cryptosporidium-positive groups at Week 1, 5 and 10, and dissimilarity between sample time points on Farm 3 (H1 vs Cp+1, p = 0.14; H5 vs Cp+5, p = 0.34; H10 vs Cp+10, p = 0.51; W1 vs W5 vs W10, p = 0.001).
