# Supplementary material for: Progression of the faecal microbiome in preweaning dairy calves that develop cryptosporidiosis
Source: Anim Microbiome. 2025 Jan 6;7:3. doi: 10.1186/s42523-024-00352-1 (PMC11706078; doi:10.1186/s42523-024-00352-1)
Supplement: Supplementary file 20 — Additional file 20: Metadata [file 42523_2024_352_MOESM20_ESM.pdf]

Progression of the faecal microbiome in preweaning dairy calves that develop cryptosporidiosis

Hares et al.

This document contains all data processing, statistical analysis, and figures generated for this study.

Mothur Sequence Processing and Diversity Data

```
#mothur 16S Pipeline

#All trimmed fastq.gz files were processed in mothur

make.file(inputdir=., type=gz, prefix=seed132)

make.contigs(file=seed132.files, processors=25)

summary.seqs(fasta=seed132.trim.contigs.fasta, count=seed132.contigs.count_table)

#Oligos file goes forward F515 sequence, reverse R806 sequence, then barcode none underneath going down the excel spreadsheet.
trim.seqs(fasta=seed132.trim.contigs.fasta, oligos=16SV4PrimerFRB.txt, pdiffs=1, checkorient=t)

summary.seqs(fasta=seed132.trim.contigs.trim.fasta, count=seed132.contigs.count_table)

screen.seqs(fasta=seed132.trim.contigs.trim.fasta, count=seed132.contigs.count_table, maxambig=0, maxlength=325, maxhomop=8)

unique.seqs(fasta=seed132.trim.contigs.trim.good.fasta, count=seed132.contigs.good.count_table)

summary.seqs(fasta=seed132.trim.contigs.trim.good.unique.fasta, count=seed132.trim.contigs.trim.good.count_table)

pcr.seqs(fasta=silva.seed_v132.align, start=11894, end=25319, keepdots=F, processors=25)

rename.file(input=silva.seed_v132.pcr.align, new=silva_v132.v4.fasta)

summary.seqs(fasta=silva_v132.v4.fasta)

align.seqs(fasta=seed132.trim.contigs.trim.good.unique.fasta, reference=silva_v132.v4.fasta)

summary.seqs(fasta=seed132.trim.contigs.trim.good.unique.align, count=seed132.trim.contigs.trim.good.count_table)

screen.seqs(fasta=seed132.trim.contigs.trim.good.unique.align, count=seed132.trim.contigs.trim.good.count_table, start=2, end=13425)

summary.seqs(fasta=seed132.trim.contigs.trim.good.unique.good.align, count=seed132.trim.contigs.trim.good.good.count_table)

filter.seqs(fasta=seed132.trim.contigs.trim.good.unique.good.align, vertical=T, trump=.)

unique.seqs(fasta=seed132.trim.contigs.trim.good.unique.good.filter.fasta, count=seed132.trim.contigs.trim.good.good.count_table)

pre.cluster(fasta=seed132.trim.contigs.trim.good.unique.good.filter.unique.fasta, count=seed132.trim.contigs.trim.good.unique.good.filter.count_table, diffs=2)

chimera.vsearch(fasta=seed132.trim.contigs.trim.good.unique.good.filter.unique.precluster.fasta, count=seed132.trim.contigs.trim.good.unique.good.filter.unique.precluster.count_table, dereplicate=t)

summary.seqs(fasta=seed132.trim.contigs.trim.good.unique.good.filter.unique.precluster.denovo.vsearch.fasta, count=seed132.trim.contigs.trim.good.unique.good.filter.unique.precluster.denovo.vsearch.count_table)

classify.seqs(fasta=seed132.trim.contigs.trim.good.unique.good.filter.unique.precluster.denovo.vsearch.fasta, count=seed132.trim.contigs.trim.good.unique.good.filter.unique.precluster.denovo.vsearch.count_table, reference=trainset18_062020.pds.fasta, taxonomy=trainset18_062020.pds.tax)

remove.lineage(fasta=seed132.trim.contigs.trim.good.unique.good.filter.unique.precluster.denovo.vsearch.fasta, count=seed132.trim.contigs.trim.good.unique.good.filter.unique.precluster.denovo.vsearch.count_table, taxonomy=seed132.trim.contigs.trim.good.unique.good.filter.unique.precluster.denovo.vsearch.pds.wang.taxonomy, taxon=Chloroplast-Mitochondria-unknown-Archaea-Eukaryota)

cluster.split(fasta=seed132.trim.contigs.trim.good.unique.good.filter.unique.precluster.denovo.vsearch.pick.fasta, count=seed132.trim.contigs.trim.good.unique.good.filter.unique.precluster.denovo.vsearch.pick.count_table, taxonomy=seed132.trim.contigs.trim.good.unique.good.filter.unique.precluster.denovo.vsearch.pds.wang.pick.taxonomy, cutoff=0.03, taxlevel=5, cluster=f, processors=25)

cluster.split(file=seed132.trim.contigs.trim.good.unique.good.filter.unique.precluster.denovo.vsearch.pick.file, processors=1)

make.shared(list=seed132.trim.contigs.trim.good.unique.good.filter.unique.precluster.denovo.vsearch.pick.opti_mcc.list, count=seed132.trim.contigs.trim.good.unique.good.filter.unique.precluster.denovo.vsearch.pick.count_table, label=0.03)

classify.otu(list=seed132.trim.contigs.trim.good.unique.good.filter.unique.precluster.denovo.vsearch.pick.opti_mcc.list, count=seed132.trim.contigs.trim.good.unique.good.filter.unique.precluster.denovo.vsearch.pick.count_table, taxonomy= seed132.trim.contigs.trim.good.unique.good.filter.unique.precluster.denovo.vsearch.pds.wang.pick.taxonomy, label=0.03)

count.groups(shared=seed132.trim.contigs.trim.good.unique.good.filter.unique.precluster.denovo.vsearch.pick.opti_mcc.shared)

make.biom(shared=seed132.trim.contigs.trim.good.unique.good.filter.unique.precluster.denovo.vsearch.pick.opti_mcc.shared, constaxonomy=seed132.trim.contigs.trim.good.unique.good.filter.unique.precluster.denovo.vsearch.pick.opti_mcc.0.03.cons.taxonomy, output=simple)

#Rarefied Alpha Diversity - Observed OTUs and Shannon Index
rarefaction.single(shared=seed132.trim.contigs.trim.good.unique.good.filter.unique.precluster.denovo.vsearch.pick.pick.opti_mcc.shared,
calc=sobs, freq=100)

summary.single(shared=seed132.trim.contigs.trim.good.unique.good.filter.unique.precluster.denovo.vsearch.pick.pick.opti_mcc.shared, subsample=19000)

#Rarefied Beta Diversity - Bray-Curtis and Jaccard PCoA
dist.shared(shared=seed132.trim.contigs.trim.good.unique.good.filter.unique.precluster.denovo.vsearch.pick.pick.opti_mcc.shared, calc=braycurtis-jclass, subsample=19000)

pcoa(phylip=final.opti_mcc.braycurtis.0.03.lt.ave.dist)

classify.otu(list=seed132.trim.contigs.trim.good.unique.good.filter.unique.precluster.denovo.vsearch.pick.opti_mcc.list, count=seed132.trim.contigs.trim.good.unique.good.filter.unique.precluster.denovo.vsearch.pick.count_table, taxonomy= seed132.trim.contigs.trim.good.unique.good.filter.unique.precluster.denovo.vsearch.pds.wang.pick.taxonomy, label=0.03, basis=sequence)

#Make a .tre file for phyloseq
get.oturep(column=seed132.trim.contigs.trim.good.unique.good.filter.unique.precluster.denovo.vsearch.pick.pick.dist, list=seed132.trim.contigs.trim.good.unique.good.filter.unique.precluster.denovo.vsearch.pick.pick.opti_mcc.list, fasta=seed132.trim.contigs.trim.good.unique.good.filter.unique.precluster.denovo.vsearch.pick.pick.fasta, count=seed132.trim.contigs.trim.good.unique.good.filter.unique.precluster.denovo.vsearch.pick.pick.count_table, cutoff=0.03)
```

R Alpha and Beta Diversity Figures

```r
library(ggplot2)
library(ggpubr)
library(tidyverse)
library(scales)
library(vegan)
library(ape)
library(phyloseq)
library(ggtext)
library(pairwiseAdonis)
library(ggmisc)
rarefy_alpha <- read_delim("mothur_alpha_diversity_output.txt")
metadata <- read_delim("16S_Metadata.txt")
rarefy_alpha_meta <- inner_join(metadata, rarefy_alpha, by="Sample_ID")
rarefy_alpha_meta$Disease_Timepoint <- factor(rarefy_alpha_meta$Disease_Timepoint, levels=c("Week 1 Control","Week 1 Cryptosporidium_Positive","Week 5 Control","Week 5 Cryptosporidium_Positive","Week 10 Control","Week 10 Cryptosporidium_Positive"))

#All analysis was repeated with Farm 2 and Farm 3 separately and together without Farm 1 to clarify the farm/antibiotic effect.

#Figure 1A - Observed OTUs Boxplot
shapiro.test((rarefy_alpha_meta$sobs))
cdt_sobs <- compare_means(sobs ~ Disease_Timepoint, rarefy_alpha_meta, method="wilcox.test", size=3, p.adjust.method = "BH")
cdt_sobs <- cdt_sobs[!(row.names(cdt_sobs) %in% c("3","4","5","6","8","9","12","13")),]
order = c(1,4,5,2,6,7,3)
cdt_sobs$order <- order
cdt_sobs <- cdt_sobs %>% arrange(order)

ad_sobs <- ggplot(rarefy_alpha_meta, aes(x=Disease_Timepoint, y=sobs, fill=Disease_Timepoint)) +
  geom_boxplot(aes(alpha = Disease_Timepoint)) +
  scale_fill_manual(values=c("dodgerblue2", "goldenrod1","dodgerblue2", "goldenrod1","dodgerblue2", "goldenrod1"), breaks =c("Week 1 Control","Week 1 Cryptosporidium_Positive","Week 5 Control","Week 5 Cryptosporidium_Positive","Week 10 Control","Week 10 Cryptosporidium_Positive")) +
  scale_x_discrete(labels = c("H1<br>(n=33)", "*Cp*+1<br>(n=32)","H5<br>(n=32)", "*Cp*+5<br>(n=32)","H10<br>(n=32)", "*Cp*+10<br>(n=30)")) +
  scale_alpha_manual(values=c(0.5,0.5,0.8,0.8,1,1)) +
  labs(x='', y= 'Observed OTUs', tag = "A") +
  geom_jitter(width=0.15, aes(fill=Disease_Timepoint), alpha=0.5, color=1, pch=21, size=3) +
  stat_summary(fun = mean, geom = "point", color = "red") +
  theme_classic() +
  theme(legend.position = "none", text = element_text(colour = "black", size=10), axis.text.x = element_markdown(colour = "black"), axis.text.y = element_text(colour = "black")) +
  stat_pvalue_manual(cdt_sobs, label="p.adj", y.position = c(450,699,852,530,530,600,600), step.increase = 0.1,inherit.aes = FALSE, size=3)
ad_sobs


#Figure 1C - Observed OTUs Loess Regression
ad_sobs_linreg <- ggplot(rarefy_alpha_meta, aes(x=Day_of_Sampling, y=sobs, fill= Disease_Status)) + geom_point(mapping = aes(x = Day_of_Sampling, y = sobs,  fill = Disease_Status), alpha=0.5, shape=21, size=2.5) + geom_smooth(mapping = aes(x = Day_of_Sampling, y = sobs,  color = Disease_Status, fill = Disease_Status), method="loess", alpha=0.2) +
  scale_y_continuous(limits = c(0,1000), expand = c(0,0)) +
  scale_x_continuous(limits = c(0,81), expand = c(0,0)) +
  scale_fill_manual(values=c("dodgerblue2", "goldenrod1"),
                    breaks=c("Control","Cryptosporidium_Positive"),
                    labels=c("H","*Cp*+")) +
  scale_color_manual(values=c("dodgerblue2", "goldenrod1"),
                    breaks=c("Control","Cryptosporidium_Positive"),
                    labels=c("H","*Cp*+")) +
  theme_bw()  +
  theme(legend.title = element_blank(), text = element_text(colour = "black", size=10), legend.text = element_markdown(colour = "black"), axis.text.y = element_text(colour = "black"), legend.position = "bottom")
ad_sobs_linreg


#Figure 1B - Shannon Index Boxplot
shapiro.test((rarefy_alpha_meta$shannon))
cdt_shan <- compare_means(shannon ~ Disease_Timepoint, rarefy_alpha_meta, method="wilcox.test", size=3, p.adjust.method = "BH")
cdt_shan <- cdt_shan[!(row.names(cdt_shan) %in% c("3","4","5","6","8","9","12","13")),]
order = c(1,4,5,2,6,7,3)
cdt_shan$order <- order
cdt_shan <- cdt_shan %>% arrange(order)

ad_shan <- ggplot(rarefy_alpha_meta, aes(x=Disease_Timepoint, y=shannon, fill=Disease_Timepoint)) +
  geom_boxplot(aes(alpha = Disease_Timepoint)) +
  scale_fill_manual(values=c("dodgerblue2", "goldenrod1","dodgerblue2", "goldenrod1","dodgerblue2", "goldenrod1"), breaks =c("Week 1 Control","Week 1 Cryptosporidium_Positive","Week 5 Control","Week 5 Cryptosporidium_Positive","Week 10 Control","Week 10 Cryptosporidium_Positive")) +
  scale_x_discrete(labels = c("H1<br>(n=33)", "*Cp*+1<br>(n=32)","H5<br>(n=32)", "*Cp*+5<br>(n=32)","H10<br>(n=32)", "*Cp*+10<br>(n=30)")) +
  scale_alpha_manual(values=c(0.5,0.5,0.8,0.8,1,1)) +
  labs(x='', y= 'Shannon Index', tag = "B") +
  geom_jitter(width=0.15, aes(fill=Disease_Timepoint), alpha=0.5, color=1, pch=21, size=3) +
  stat_summary(fun = mean, geom = "point", color = "red")+
  theme_classic()  +
  theme(legend.position = "none", text = element_text(colour = "black", size=10), axis.text.x = element_markdown(colour = "black"), axis.text.y = element_text(colour = "black")) +
  stat_pvalue_manual(cdt_shan, label="p.adj", y.position = c(4,4.48,4.97,3.58,3.6,3.6,3.6), step.increase = 0.1,inherit.aes = FALSE, size=3)
ad_shan

#Figure 1D - Shannon Index Loess Regression
ad_shan_linreg <- ggplot(rarefy_alpha_meta, aes(x=Day_of_Sampling, y=shannon, fill=Disease_Status)) +
  geom_point(mapping = aes(x = Day_of_Sampling, y = shannon,  fill = Disease_Status), alpha=0.5, shape=21, size=2.5) +
  geom_smooth(mapping = aes(x = Day_of_Sampling, y = shannon,  color = Disease_Status, fill = Disease_Status), method="loess", alpha=0.2) +
  scale_y_continuous(limits = c(0,5.5), expand = c(0,0)) +
  scale_x_continuous(limits = c(0,81), expand = c(0,0)) +
  scale_fill_manual(values=c("dodgerblue2", "goldenrod1"),
                    breaks=c("Control","Cryptosporidium_Positive"),
                    labels=c("H","*Cp*+")) +
  scale_color_manual(values=c("dodgerblue2", "goldenrod1"),
                    breaks=c("Control","Cryptosporidium_Positive"),
                    labels=c("H","*Cp*+")) +
  labs(x='Day of Sampling', y= 'Shannon Index', tag = "D") +
  theme_bw()  +
  theme(legend.title = element_blank(), text = element_text(colour = "black", size=10), legend.text = element_markdown(colour = "black"), axis.text.y = element_text(colour = "black"), legend.position = "bottom")
ad_shan_linreg

#Alpha Diversity Estimated Marginal Means
library(glmmTMB)
library(DHARMa)
library(emmeans)
library(car)
library(tidyverse)
library(ggtext)
library(ggpubr)
library(moments)

rarefy_alpha <- read.delim("mothur_alpha_diversity_output.txt")
metadata <- read.delim("16S_Metadata.txt")
rarefy_alpha_meta <- inner_join(metadata, rarefy_alpha, by="Sample_ID")

shapiro.test((rarefy_alpha_meta$shannon))
hist(rarefy_alpha_meta$shannon)
skewness(rarefy_alpha_meta$shannon, na.rm = TRUE)
rarefy_alpha_meta$shannon <- log10(max(rarefy_alpha_meta$shannon+1) - rarefy_alpha_meta$shannon)

summary(Shannon <- glmmTMB(shannon ~
                  Disease_Status +
                  Farm +
                  Diatrim +
                  Synulox +
                  Draaxin +
                  Halocur +
                  Day_of_Sampling +
                  Disease_Status*Day_of_Sampling +
                  (1|Study_ID),
                data = rarefy_alpha_meta,
                family = gaussian))

# check model fit - check the residuals, ideally want pvals to be non-significant
testResiduals(Shannon)
testQuantiles(Shannon)

# get global p values for co-variates
car::Anova(Shannon)

# post-hoc pairwise analysis
x <- emmeans(Shannon, specs = pairwise ~ Disease_Status*Day_of_Sampling)
x

# plot estimated marginal means
plot(x, comparisons = TRUE) +
  theme_bw() +
  labs(title = "Estimated Marginal Means of Shannon Index") +
  scale_y_discrete(labels= c("H","*Cp*+")) +
  xlab("Emmean") +
  theme(text = element_text(size = 11),
        legend.title = element_blank(),
        axis.text.y = element_markdown(),
        axis.title = element_blank())

# fit richness model
summary(Richness <-
          glmmTMB(sobs ~
                  Disease_Status +
                  Farm +
                  Diatrim +
                  Synulox +
                  Draaxin +
                  Halocur +
                  Day_of_Sampling +
                  Disease_Status*Day_of_Sampling +
                  (1|Study_ID),
                data = rarefy_alpha_meta,
                family = gaussian()))

testResiduals(Richness)

car::Anova(Richness)

emm = emmeans(Richness, specs = pairwise ~ Disease_Status*Day_of_Sampling)
emm

# plot estimated marginal means
plot(emm, comparisons = TRUE) +
  theme_bw() +
  labs(title = "Estimated Marginal Means of Observed OTUs") +
  scale_y_discrete(labels= c("H","*Cp*+")) +
  xlab("Emmean") +
  theme(text = element_text(size = 11),
        legend.title = element_blank(),
        axis.text.y = element_markdown(),
        axis.title = element_blank())

#Figure 2A - Bray Curtis Beta Diversity PCoA Plot
pcoa_bc<-read.table(file="seed132.trim.contigs.trim.good.unique.good.filter.unique.precluster.denov.vsearch.pick.pick.opti_mcc.braycurtis.0.03.lt.ave.pcoa.axes", header=T)
metadata_g <- metadata %>% dplyr::rename(group = Sample_ID)
rarefy_pcoa_bc_meta <- inner_join(metadata_g, pcoa_bc, by="group")
centroid <- rarefy_pcoa_bc_meta %>%
  dplyr::group_by(Disease_Timepoint) %>%
  dplyr::summarize(axis1 = mean(axis1),
            axis2 = mean(axis2), .groups="drop")

star <- rarefy_pcoa_bc_meta %>%
  dplyr::group_by(Disease_Timepoint) %>%
  dplyr::mutate(centroid1 = mean(axis1),
         centroid2 = mean(axis2)) %>%
  ungroup

bc_pcoa <- ggplot(star, aes(x = axis1, xend=centroid1, y = axis2, yend=centroid2, colour = Disease_Timepoint,  fill = Disease_Timepoint)) +
  geom_point(aes(fill=Disease_Timepoint), color=1, pch=21, size=3) +
  geom_segment() +
  geom_point(data = centroid, mapping= aes(x = axis1, y = axis2), colour = "black", fill = "yellow", shape = 21, size=4, show.legend=FALSE, inherit.aes = FALSE) +
  stat_ellipse(aes(fill=Disease_Timepoint, colour=Disease_Timepoint), geom = "polygon", type = "norm", alpha=0.1, show.legend=FALSE) +
  labs(x="PC1 (13.5%)", y="PC2 (6.8%)", fill = "Disease_Timepoint", colour = "Disease_Timepoint", tags = "A") +

  scale_colour_manual(values = c("dodgerblue2", "goldenrod1","darkorchid","chartreuse3","deeppink1","cyan3"),
                      labels = c("H1<br>(n=33)", "*Cp*+1<br>(n=32)","H5<br>(n=32)", "*Cp*+5<br>(n=32)","H10<br>(n=32)", "*Cp*+10<br>(n=30)")) +
  scale_fill_manual(values = c("dodgerblue2", "goldenrod1","darkorchid","chartreuse3","deeppink1","cyan3"),
                    labels = c("H1<br>(n=33)", "*Cp*+1<br>(n=32)","H5<br>(n=32)", "*Cp*+5<br>(n=32)","H10<br>(n=32)", "*Cp*+10<br>(n=30)")) +
  theme_bw() +
  theme(axis.text.y = element_text(colour = "black", size = 11),
        axis.text.x = element_text(colour = "black", size = 11),
        legend.text = element_markdown(size = 8, colour = "black"),
        legend.position = "bottom", axis.title.y = element_text(size = 12),
        axis.title.x = element_text(size = 12, colour = "black"),
        legend.title = element_blank(),
        panel.background = element_blank(), panel.border = element_rect(colour = "black", fill = NA),
        legend.key=element_blank(),
        plot.caption = element_text(hjust=0),
        plot.caption.position = "plot") +
        guides(color = guide_legend(nrow = 1))
bc_pcoa

#Bray Curtis PERMANOVA Statistical Tests
set.seed(1)
library(pairwiseAdonis)
bcdistance <- read_tsv("seed132-braycurtis.0.03.square.ave.dist", skip=1, col_names = FALSE)
colnames(bcdistance) <- c("Sample_ID", bcdistance$X1)
bcdistance$Sample_ID <- as.character(bcdistance$Sample_ID)
t_metadata <- as_tibble(metadata)
t_metadata$Sample_ID <- as.character(t_metadata$Sample_ID)
t_metadata$Calf_Tag_ID <- as.character(t_metadata$Calf_Tag_ID)
t_metadata$Farm <- as.character(t_metadata$Farm)
bc_meta_dist <- inner_join(t_metadata,bcdistance, by="Sample_ID")
bc_all_dist <- bc_meta_dist %>%
  select(all_of(.[["Sample_ID"]])) %>%
  as.dist()

bc_PP <- pairwise.adonis2(bc_all_dist~Disease_Timepoint, data=bc_meta_dist, p_adjust_m = "BH")
bc_pairwise_p <- numeric()
bc_pairwise_p["W1CtrlvsCP"] <- bc_PP[["Week 1 Cryptosporidium_Positive_vs_Week 1 Control"]][["Pr(>F)"]][1]
bc_pairwise_p["W5CtrlvsCP"] <- bc_PP[["Week 5 Cryptosporidium_Positive_vs_Week 5 Control"]][["Pr(>F)"]][1]
bc_pairwise_p["W10CtrlvsCP"] <- bc_PP[["Week 10 Cryptosporidium_Positive_vs_Week 10 Control"]][["Pr(>F)"]][1]
bc_pairwise_p["W1CtrlvsW5Ctrl"] <- bc_PP[["Week 1 Control_vs_Week 5 Control"]][["Pr(>F)"]][1]
bc_pairwise_p["W1CtrlvsW10Ctrl"] <- bc_PP[["Week 1 Control_vs_Week 10 Control"]][["Pr(>F)"]][1]
bc_pairwise_p["W5CtrlvsW10Ctrl"] <- bc_PP[["Week 5 Control_vs_Week 10 Control"]][["Pr(>F)"]][1]
bc_pairwise_p["W1CPvsW5CP"] <- bc_PP[["Week 1 Cryptosporidium_Positive_vs_Week 5 Cryptosporidium_Positive"]][["Pr(>F)"]][1]
bc_pairwise_p["W1CPvsW10CP"] <- bc_PP[["Week 1 Cryptosporidium_Positive_vs_Week 10 Cryptosporidium_Positive"]][["Pr(>F)"]][1]
bc_pairwise_p["W5CPvsW10CP"] <- bc_PP[["Week 5 Cryptosporidium_Positive_vs_Week 10 Cryptosporidium_Positive"]][["Pr(>F)"]][1]
bc_pairwise_p

bc <- betadisper(bc_all_dist, bc_meta_dist$Disease_Timepoint)
permutest(bc, pairwise = TRUE)

#Figure 2B - Jaccard Beta Diversity PCoA Plot
pcoa_j <-read.table(file="seed132.trim.contigs.trim.good.unique.good.filter.unique.precluster.denovo.vsearch.pick.pick.opti_mcc.jclass.0.03.lt.ave.pcoa.axes", header=T)
rarefy_pcoa_j_meta <- inner_join(metadata_g, pcoa_j, by="group")
jcentroid <- rarefy_pcoa_j_meta %>%
  dplyr::group_by(Disease_Timepoint) %>%
  dplyr::summarize(axis1 = mean(axis1),
            axis2 = mean(axis2), .groups="drop")

jstar <- rarefy_pcoa_j_meta %>%
  dplyr::group_by(Disease_Timepoint) %>%
  dplyr::mutate(centroid1 = mean(axis1),
         centroid2 = mean(axis2)) %>%
  ungroup

j_pcoa <- ggplot(jstar, aes(x = axis1, xend=centroid1, y = axis2, yend=centroid2, colour = Disease_Timepoint,  fill = Disease_Timepoint)) +
  geom_point(aes(fill=Disease_Timepoint), color=1, pch=21, size=3) +
  geom_segment() +
  geom_point(data = jcentroid, mapping= aes(x = axis1, y = axis2), colour = "black", fill = "yellow", shape = 21, size=4, show.legend=FALSE, inherit.aes = FALSE) +
  stat_ellipse(aes(fill=Disease_Timepoint, colour=Disease_Timepoint), geom = "polygon", type = "norm", alpha=0.1, show.legend=FALSE) +
  labs(x="PC1 (10.2%)", y="PC2 (4.5%)", fill = "Disease_Timepoint", colour = "Disease_Timepoint", tags = "B") +
  scale_colour_manual(values = c("dodgerblue2", "goldenrod1","darkorchid","chartreuse3","deeppink1","cyan3"),
                      labels = c("H1<br>(n=33)", "*Cp*+1<br>(n=32)","H5<br>(n=32)", "*Cp*+5<br>(n=32)","H10<br>(n=32)", "*Cp*+10<br>(n=30)")) +
  scale_fill_manual(values = c("dodgerblue2", "goldenrod1","darkorchid","chartreuse3","deeppink1","cyan3"),
                    labels = c("H1<br>(n=33)", "*Cp*+1<br>(n=32)","H5<br>(n=32)", "*Cp*+5<br>(n=32)","H10<br>(n=32)", "*Cp*+10<br>(n=30)")) +
  theme_bw() +
  theme(axis.text.y = element_text(colour = "black", size = 11),
        axis.text.x = element_text(colour = "black", size = 11),
        legend.text = element_markdown(size = 8, colour = "black"),
        legend.position = "bottom", axis.title.y = element_text(size = 12),
        axis.title.x = element_text(size = 12, colour = "black"),
        legend.title = element_blank(),
        panel.background = element_blank(), panel.border = element_rect(colour = "black", fill = NA),
        legend.key=element_blank()) +
  guides(color = guide_legend(nrow = 1))
j_pcoa

library(gridExtra)
grid.arrange(bc_pcoa,j_pcoa)

#Jaccard PERMANOVA Statistical Tests
library(pairwiseAdonis)
jdistance <- read_tsv("seed132.trim.contigs.trim.good.unique.good.filter.unique.precluster.denovo.vsearch.pick.pick.opti_mcc.jclass.0.03.square.ave.dist", skip=1, col_names = FALSE)
colnames(jdistance) <- c("Sample_ID", jdistance$X1)
jdistance$Sample_ID <- as.character(jdistance$Sample_ID)
t_metadata <- as_tibble(metadata)
t_metadata$Sample_ID <- as.character(t_metadata$Sample_ID)
t_metadata$Calf_Tag_ID <- as.character(t_metadata$Calf_Tag_ID)
t_metadata$Farm <- as.character(t_metadata$Farm)
j_meta_dist <- inner_join(t_metadata,jdistance, by="Sample_ID")
j_all_dist <- j_meta_dist %>%
  select(all_of(.[["Sample_ID"]])) %>%
  as.dist()

j_PP <- pairwise.adonis2(j_all_dist~Disease_Timepoint, data=j_meta_dist, p_adjust_m = "BH")
j_pairwise_p <- numeric()
j_pairwise_p["W1CtrlvsCP"] <- j_PP[["Week 1 Cryptosporidium_Positive_vs_Week 1 Control"]][["Pr(>F)"]][1]
j_pairwise_p["W5CtrlvsCP"] <- j_PP[["Week 5 Cryptosporidium_Positive_vs_Week 5 Control"]][["Pr(>F)"]][1]
j_pairwise_p["W10CtrlvsCP"] <- j_PP[["Week 10 Cryptosporidium_Positive_vs_Week 10 Control"]][["Pr(>F)"]][1]
j_pairwise_p["W1CtrlvsW5Ctrl"] <- j_PP[["Week 1 Control_vs_Week 5 Control"]][["Pr(>F)"]][1]
j_pairwise_p["W1CtrlvsW10Ctrl"] <- j_PP[["Week 1 Control_vs_Week 10 Control"]][["Pr(>F)"]][1]
j_pairwise_p["W5CtrlvsW10Ctrl"] <- j_PP[["Week 5 Control_vs_Week 10 Control"]][["Pr(>F)"]][1]
j_pairwise_p["W1CPvsW5CP"] <- j_PP[["Week 1 Cryptosporidium_Positive_vs_Week 5 Cryptosporidium_Positive"]][["Pr(>F)"]][1]
j_pairwise_p["W1CPvsW10CP"] <- j_PP[["Week 1 Cryptosporidium_Positive_vs_Week 10 Cryptosporidium_Positive"]][["Pr(>F)"]][1]
j_pairwise_p["W5CPvsW10CP"] <- j_PP[["Week 5 Cryptosporidium_Positive_vs_Week 10 Cryptosporidium_Positive"]][["Pr(>F)"]][1]
j_pairwise_p

jac <- betadisper(j_all_dist, j_meta_dist$Disease_Timepoint)
permutest(jac, pairwise = TRUE)
```

Rarefaction Curves

```r
library(tidyverse)
library(vegan)
library(ggtext)
library(markdown)
#Figure S5: Rarefaction Curve
set.seed(1)
#Import rarefied data from mothur
rarefy <- read_tsv(file="seed132.trim.contigs.trim.good.unique.good.filter.unique.precluster.denovo.vsearch.pick.pick.opti_mcc.groups.rarefaction") %>%
  select(-contains("lci-"), -contains("hci-")) %>%
  pivot_longer(cols=c(-numsampled), names_to='Sample_ID', values_to='sobs') %>%
  mutate(Sample_ID=str_replace_all(Sample_ID, pattern="0.03-", replacement="")) %>%
  drop_na()

metadata<-read_delim("16S_Metadata.txt")
metadata <- metadata %>%
  mutate(Sample_ID = as.character(Sample_ID))

metadata_rarefy <- inner_join(metadata, rarefy)

ggplot(metadata_rarefy, aes(x=numsampled, y=sobs, group=Sample_ID, color=Disease_Timepoint)) +
  geom_line(linewidth=0.6) +
  geom_vline(xintercept=19000, color="gray", size=1) +
  scale_color_manual(name=NULL,
                     values=c("dodgerblue2", "goldenrod1","darkorchid","chartreuse3","deeppink1","cyan3"),
                     breaks=c("Week 1 Control", "Week 1 Cryptosporidium_Positive","Week 5 Control", "Week 5 Cryptosporidium_Positive","Week 10 Control", "Week 10 Cryptosporidium_Positive"),
                     labels=c("H1 (n=33)", "*Cp*+1 (n=32)","H5 (n=32)", "*Cp*+5 (n=32)","H10 (n=32)", "*Cp*+10 (n=30)")) +
  coord_cartesian(xlim=c(0,60000), ylim=c(0,1500)) +
  labs(title=" ",
       x="Rarefaction Depth",
       y="Observed OTUs") +
  theme_classic() +
  theme(legend.text = element_markdown(), legend.position = "bottom") +
  guides(color = guide_legend(nrow = 1))
```

R Taxa Relative Abundance Figures

```r
library(ggplot2)
library(vegan)
library(plyr)
library(dplyr)
library(scales)
library(grid)
library(reshape2)
library(cowplot)
library(phyloseq)
library(ape)
library(tidyverse)
library(readxl)
library(ggtext)
library(RColorBrewer)
library(egg)
library(base)
library(ggpubr)
library(glue)
library(markdown)

#Import mothur output files
sharedfile = "seed132.trim.contigs.trim.good.unique.good.filter.unique.precluster.denovo.vsearch.pick.pick.opti_mcc.shared"
taxfile = "seed132.trim.contigs.trim.good.unique.good.filter.unique.precluster.denovo.vsearch.pick.pick.opti_mcc.0.03.cons.taxonomy"
treefile <- "seed132.trim.contigs.trim.good.unique.good.filter.unique.precluster.denovo.vsearch.pick.pick.opti_mcc.0.03.rep.otu.phylip.tre"
mapfile = "16S_Metadata.txt"

mothur_data <- import_mothur(mothur_shared_file = sharedfile,
                             mothur_constaxonomy_file = taxfile,
                             mothur_tree_file = treefile)

map <- read.delim(mapfile)
head(map)
set.seed(1)
# transform dataframe into phyloseq class object.
map <- sample_data(map)
# Assign rownames to be Sample ID's
rownames(map) <- map$group
#checking the object "map"
str(map)
# Merge mothurdata object with sample metadata
mothur_merge <- merge_phyloseq(mothur_data, map)
# showing the object mothur_merge
mothur_merge
head(mothur_merge@tax_table, n=10)
colnames(tax_table(mothur_merge)) <- c("Domain", "Phylum", "Class","Order", "Family", "Genus")
colnames(tax_table(mothur_merge))
# create object calf_data
calf_data <- mothur_merge %>%
  subset_taxa(
    Domain == "Bacteria" &
      Family  != "mitochondria" &
      Class   != "Chloroplast")

# Make a data frame with a column for the read counts of each sample
sample_sum_df <- data.frame(sum = sample_sums(calf_data))

# Figure S4: Histogram of sample sequencing depth distribution
ggplot(sample_sum_df, aes(x = sum)) +
  geom_histogram(color = "black", fill = "dodgerblue2", binwidth = 1000) +
  scale_y_continuous(expand = c(0,0)) +
  xlab("Read Counts") +
  ylab("Number of Samples") +
  theme_classic() +
  theme(axis.text = element_text(colour = "black"), axis.title = element_text(colour = "black"))

#Rarefaction of data
calf_scaled <- rarefy_even_depth(calf_data,sample.size=19000, replace=FALSE, rngseed = 1)

#Figure 3A - Phylum Relative Abundance Stacked Barchart
cdata_phylum <- calf_scaled %>%
  tax_glom(taxrank = "Phylum") %>%                     # agglomerate at phylum level
  transform_sample_counts(function(x) {x/sum(x)} ) %>% # Transform to rel. abundance
  psmelt()                                             # Melt to long format

phy_rel_abund <- cdata_phylum %>%
  dplyr::group_by(Disease_Timepoint, Phylum, Sample_Group,Disease_Status) %>%
  dplyr::summarize(Abundance = sum(Abundance), .groups="drop") %>%
  dplyr::group_by(Disease_Timepoint, Phylum, Sample_Group,Disease_Status) %>%
  dplyr::summarize(mean_rel_abund = 100*mean(Abundance), .groups="drop") %>%
  dplyr::mutate(Phylum = str_replace(Phylum,"(.*)_unclassified", "Unclassified \\1"),
         Phylum = str_replace_all(Phylum,"_"," "))

phy_taxon_pool <- phy_rel_abund %>%
  dplyr::group_by(Phylum) %>%
  dplyr::summarize(pool = mean(mean_rel_abund) < 0.5,
            mean = mean(mean_rel_abund),
            .groups="drop")

phy <- dplyr::inner_join(phy_rel_abund, phy_taxon_pool, by="Phylum") %>%
  dplyr::mutate(Phylum= if_else(pool, "Other", Phylum)) %>%
  dplyr::group_by(Phylum,Disease_Timepoint) %>%
  dplyr::summarize(mean_rel_abund = sum(mean_rel_abund),
            mean = min(mean),
            .groups="drop") %>%
  dplyr::mutate(Phylum = factor(Phylum),
         Phylum = fct_reorder(Phylum, mean, .desc=TRUE),
         Phylum = fct_shift(Phylum, n=1))

phy$Disease_Timepoint <- factor(phy$Disease_Timepoint, levels=c("Week 1 Control","Week 1 Cryptosporidium_Positive","Week 5 Control","Week 5 Cryptosporidium_Positive","Week 10 Control","Week 10 Cryptosporidium_Positive"))

phylum_colors <- c("dodgerblue2", "goldenrod1","darkorchid","chartreuse3","deeppink1","darkgrey")

ggplot(phy, aes(x = Disease_Timepoint, y = mean_rel_abund, fill=Phylum)) +
  geom_bar(position = "fill", stat = "identity", width=0.95) +
  scale_y_continuous(expand = c(0,0), breaks = c(0.00,0.25,0.50,0.75,1.00),
                     labels = c("0","25", "50","75","100")) +
  scale_fill_manual(values = phylum_colors,
                    name=NULL,
                    breaks=c("Actinobacteria","Bacteroidetes", "Firmicutes","Fusobacteria","Proteobacteria","Other"),
                    labels=c("*Actinobacteria*","*Bacteroidetes*","*Firmicutes*","*Fusobacteria*","*Proteobacteria*","Other")) +
  scale_x_discrete(expand = c(0,0),
    breaks = c("Week 1 Control", "Week 1 Cryptosporidium_Positive","Week 5 Control", "Week 5 Cryptosporidium_Positive","Week 10 Control", "Week 10 Cryptosporidium_Positive"),
    labels = c("H1<br>(n=33)", "*Cp*+1<br>(n=32)","H5<br>(n=32)", "*Cp*+5<br>(n=32)","H10<br>(n=32)", "*Cp*+10<br>(n=30)"),
      drop = FALSE)  +
  theme_classic() +
    theme(axis.title.x = element_blank(),
        axis.text.x=element_markdown(colour="black", size=8),
        axis.text.y=element_text(colour="black"),
        legend.title = element_blank(),
        legend.text = element_markdown(size=8)) +
  guides(fill = guide_legend(keywidth = 1, keyheight = 1)) +
  ylab("Relative Abundance (%)") +
  labs(tag = "A")

#Figure 3B - Genera Relative Abundance Stacked Barchart
cdata_gen <- calf_scaled %>%
  tax_glom(taxrank = "Genus") %>%                      # agglomerate at genus level
  transform_sample_counts(function(x) {x/sum(x)} ) %>% # Transform to rel. abundance
  psmelt()                                             # Melt to long format

gen_rel_abund <- cdata_gen %>%
  dplyr::group_by(Disease_Timepoint, Sample_ID, Genus, Sample_Group) %>%
  dplyr::summarize(Abundance = sum(Abundance), .groups="drop") %>%
  dplyr::group_by(Disease_Timepoint, Genus, Sample_Group) %>%
  dplyr::summarize(mean_rel_abund = 100*mean(Abundance), .groups="drop") %>%
  dplyr::mutate(Genus = str_replace(Genus,"(.*)_unclassified", "Unclassified \\1"),
         Genus = str_replace_all(Genus,"_"," "))

gen_taxon_pool <- gen_rel_abund %>%
  dplyr::group_by(Genus) %>%
  dplyr::summarize(pool = mean(mean_rel_abund) < 2,
            mean = mean(mean_rel_abund),
            .groups="drop")

gen <- dplyr::inner_join(gen_rel_abund, gen_taxon_pool, by="Genus") %>%
  dplyr::mutate(Genus= if_else(pool, "Other", Genus)) %>%
  dplyr::group_by(Disease_Timepoint, Genus, Sample_Group) %>%
  dplyr::summarize(mean_rel_abund = sum(mean_rel_abund),
            mean = min(mean),
            .groups="drop")

gen$Disease_Timepoint <- factor(gen$Disease_Timepoint, levels=c("Week 1 Control","Week 1 Cryptosporidium_Positive","Week 5 Control","Week 5 Cryptosporidium_Positive","Week 10 Control","Week 10 Cryptosporidium_Positive"))

genus_colors <- c("dodgerblue2", "goldenrod1","darkorchid","chartreuse3","darkorange","deeppink1", "cyan","magenta", "maroon","pink","forestgreen","mediumpurple1","yellow","blue","olivedrab1","darkgrey")

ggplot(gen, aes(x = Disease_Timepoint, y = mean_rel_abund, fill=Genus)) +
  geom_bar(position = "fill", stat = "identity", width=0.95) +
  scale_y_continuous(expand = c(0,0), breaks = c(0.00,0.25,0.50,0.75,1.00),
                     labels = c("0","25", "50","75","100")) +
  scale_fill_manual(values = genus_colors,
                    name=NULL,
                    breaks = c("Bacteroides","Bifidobacterium","Blautia","Clostridium sensu stricto","Collinsella","Escherichia/Shigella","Faecalibacterium","Megamonas","Phascolarctobacterium","Phocaeicola","Prevotella","Veillonella","Unclassified Clostridiales","Unclassified Lachnospiraceae","Unclassified Ruminococcaceae","Other"),
                    labels = c("*Bacteroides*","*Bifidobacterium*","*Blautia*","*Clostridium sensu stricto*","*Collinsella*","*Escherichia/Shigella*","*Faecalibacterium*","*Megamonas*","*Phascolarctobacterium*","*Phocaeicola*","*Prevotella*","*Veillonella*","Uncl. *Clostridiales*","Uncl. *Lachnospiraceae*","Uncl. *Ruminococcaceae*","Other")) +
  scale_x_discrete(expand = c(0,0),
                   breaks = c("Week 1 Control", "Week 1 Cryptosporidium_Positive","Week 5 Control", "Week 5 Cryptosporidium_Positive","Week 10 Control", "Week 10 Cryptosporidium_Positive"),
                   labels = c("H1<br>(n=33)", "*Cp*+1<br>(n=32)","H5<br>(n=32)", "*Cp*+5<br>(n=32)","H10<br>(n=32)", "*Cp*+10<br>(n=30)"), drop=FALSE)  +
  theme_classic() +
  theme(axis.title.x = element_blank(),
        axis.text.x=element_markdown(colour="black",size=8),
        plot.margin=margin(10,10,10,10),
        axis.text.y=element_text(colour="black"),
        legend.title = element_blank(),
        legend.text = element_markdown(size=8)) +
  guides(fill = guide_legend(keywidth = 1, keyheight = 1, ncol=1)) +
  ylab("Relative Abundance (%)") +
  labs(tag = "B")

#Figure 3C - Core Microbiome Venn Diagrams
library(eulerr)
library(microbiome)
library(microbiomeutilities)
pseq <- calf_scaled
table(meta(pseq)$Disease_Status, meta(pseq)$Sample_Group)
#Week 1
ps.w1 <- subset_samples(pseq, Sample_Group == "Week 1")
print(ps.w1)
ps.w1 <- prune_taxa(taxa_sums(ps.w1) > 0, ps.w1)
print(ps.w1)
ps.w1.rel <- microbiome::transform(ps.w1, "compositional")
taxa_names(ps.w1.rel)[1:2]
ps.w1.rel.gen <- aggregate_taxa(ps.w1.rel, "Genus")
taxa_names(ps.w1.rel.gen)[1:10]
disease_w1 <- unique(as.character(meta(ps.w1.rel.gen)$Disease_Status))
print(disease_w1)
list_core_w1 <- c()
for (n in disease_w1){ # for each variable n in Disease_Timepoint
  #print(paste0("Identifying Core Taxa for ", n))

  ps.sub <- subset_samples(ps.w1.rel.gen, Disease_Status == n) # Choose sample from Disease_Timepoint by n

  core_m <- core_members(ps.sub, # ps.sub is phyloseq selected with only samples from g
                         detection = 0.0001, # 0.01% abundance in at least 80% samples
                         prevalence = 0.8)

  print(paste0("No. of core taxa in ", n, " : ", length(core_m))) # print core taxa identified in each Disease_Timepoint.
  list_core_w1[[n]] <- core_m # add to a list core taxa for each group.
  #print(list_core)
}
print(list_core_w1)
# Specify colors and plot venn
# supplying colors in the order they appear in list_core
display_venn_w1 <- function(x, ...){
  library(VennDiagram)
  grid.newpage()
  venn_object <- venn.diagram(list_core_w1, filename = NULL, ...)
  grid.draw(venn_object)
}
display_venn_w1(x,
              disable.logging = TRUE,
              category.names = c((expression(italic("Cp+")*1)),"H1"),
              col=c("goldenrod3","dodgerblue3"),
              fill = c("goldenrod1","dodgerblue2"),
              imagetype="png" ,
              compression = "lzw",
              lwd = 3,
              cex = 1.5,
              main.pos=c(0.5,0.9),
              main.fontfamily = "sans",
              main.cex = 1.5,
              fontfamily = "sans",
              cat.cex = 1.5,
              cat.default.pos = "outer",
              cat.pos = c(10, -10),
              cat.dist = c(0.05, 0.05),
              cat.fontfamily = "sans",
              scaled = FALSE,
              rotation.degree=180)


df_core_w1 <- as.data.frame(do.call(cbind, list_core_w1))
library(xlsx)
write.xlsx (x = as.list.data.frame(df_core_w1), file = "core_microbiome_w1.xlsx")


#Week 5
ps.w5 <- subset_samples(pseq, Sample_Group == "Week 5")
print(ps.w5)
ps.w5 <- prune_taxa(taxa_sums(ps.w5) > 0, ps.w5)
print(ps.w5)
ps.w5.rel <- microbiome::transform(ps.w5, "compositional")
taxa_names(ps.w5.rel)[1:2]
ps.w5.rel.gen <- aggregate_taxa(ps.w5.rel, "Genus")
taxa_names(ps.w5.rel.gen)[1:10]
disease_w5 <- unique(as.character(meta(ps.w5.rel.gen)$Disease_Status))
print(disease_w5)
list_core_w5 <- c()
for (n in disease_w5){ # for each variable n in Disease_Timepoint
  #print(paste0("Identifying Core Taxa for ", n))

  ps.sub <- subset_samples(ps.w5.rel.gen, Disease_Status == n) # Choose sample from Disease_Timepoint by n

  core_m <- core_members(ps.sub, # ps.sub is phyloseq selected with only samples from g
                         detection = 0.0001, # 0.0001 in at least 80% samples
                         prevalence = 0.8)

  print(paste0("No. of core taxa in ", n, " : ", length(core_m))) # print core taxa identified in each Disease_Timepoint.
  list_core_w5[[n]] <- core_m # add to a list core taxa for each group.
  #print(list_core)
}
print(list_core_w5)
# Specify colors and plot venn
# supplying colors in the order they appear in list_core
display_venn_w5 <- function(x, ...){
  library(VennDiagram)
  grid.newpage()
  venn_object <- venn.diagram(list_core_w5, filename = NULL, ...)
  grid.draw(venn_object)
}
display_venn_w5(x,
              disable.logging = TRUE,
              category.names = c("H5",expression(italic("Cp+")*5)),
              col=c("darkorchid4","chartreuse4"),
              fill = c("darkorchid","chartreuse3"),
              imagetype="png" ,
              base_height = 480 ,
              base_width = 480 ,
              resolution = 300,
              compression = "lzw",
              lwd = 3,
              cex = 1.5,
              main.pos=c(0.5,0.9),
              main.fontfamily = "sans",
              main.cex = 1.5,
              fontfamily = "sans",
              cat.cex = 1.5,
              cat.default.pos = "outer",
              cat.pos = c(-10, 10),
              cat.dist = c(0.05, 0.05),
              cat.fontfamily = "sans",
              scaled = FALSE,
              rotation.degree=180)
df_core_w5 <- as.data.frame(do.call(cbind, list_core_w5))
write.xlsx (x = as.list.data.frame(df_core_w5), file = "core_microbiome_w5.xlsx")

#Week 10
ps.w10 <- subset_samples(pseq, Sample_Group == "Week 8")
print(ps.w10)
ps.w10 <- prune_taxa(taxa_sums(ps.w10) > 0, ps.w10)
print(ps.w10)
ps.w10.rel <- microbiome::transform(ps.w10, "compositional")
taxa_names(ps.w10.rel)[1:2]
ps.w10.rel.gen <- aggregate_taxa(ps.w10.rel, "Genus")
taxa_names(ps.w10.rel.gen)[1:10]
disease_w10 <- unique(as.character(meta(ps.w10.rel.gen)$Disease_Status))
print(disease_w10)
list_core_w10 <- c()
for (n in disease_w10){ # for each variable n in Disease_Timepoint
  #print(paste0("Identifying Core Taxa for ", n))

  ps.sub <- subset_samples(ps.w10.rel.gen, Disease_Status == n) # Choose sample from Disease_Timepoint by n

  core_m <- core_members(ps.sub, # ps.sub is phyloseq selected with only samples from g
                         detection = 0.0001, # 0.0001 in at least 80% samples
                         prevalence = 0.8)

  print(paste0("No. of core taxa in ", n, " : ", length(core_m))) # print core taxa identified in each Disease_Timepoint.
  list_core_w10[[n]] <- core_m # add to a list core taxa for each group.
  #print(list_core)
}
print(list_core_w10)
# Specify colors and plot venn
# supplying colors in the order they appear in list_core
display_venn_w10 <- function(x, ...){
  library(VennDiagram)
  grid.newpage()
  venn_object <- venn.diagram(list_core_w10, filename = NULL, ...)
  grid.draw(venn_object)
}
display_venn_w10(x,
                disable.logging = TRUE,
                category.names = c((expression(italic("Cp+")*10)),"H10"),
                col=c("deeppink3","cyan4"),
                fill = c("deeppink1","cyan3"),
                imagetype="png" ,
                base_height = 480 ,
                base_width = 480 ,
                resolution = 300,
                compression = "lzw",
                lwd = 3,
                cex = 1.5,
                main.pos=c(0.5,0.9),
                main.fontfamily = "sans",
                main.cex = 1.5,
                fontfamily = "sans",
                cat.cex = 1.5,
                cat.default.pos = "outer",
                cat.pos = c(0, 0),
                cat.dist = c(0.05, 0.09),
                cat.fontfamily = "sans",
                scaled = FALSE,
                rotation.degree=180,
                margin = 0.175)
df_core_w10 <- as.data.frame(do.call(cbind, list_core_w10))
write.xlsx (x = as.list.data.frame(df_core_w10), file = "core_microbiome_w10.xlsx")

#Figure 4 - Top 30 Most Abundant Taxa Boxplots
cdata_gen <- calf_scaled %>%
  tax_glom(taxrank = "Genus") %>%                      # agglomerate at phylum level
  transform_sample_counts(function(x) {x/sum(x)} ) %>% # Transform to rel. abundance
  psmelt() #%>%                                        # Melt to long format

gen_rel_abund <- cdata_gen %>%
  dplyr::group_by(Disease_Timepoint, Study_ID, Genus, Disease_Status) %>%
  dplyr::summarize(Abundance = sum(Abundance), .groups="drop") %>%
  dplyr::group_by(Disease_Timepoint, Study_ID, Genus, Disease_Status) %>%
  dplyr::summarize(mean_rel_abund = 100*mean(Abundance), .groups="drop") %>%
  dplyr::mutate(Genus = str_replace(Genus,"(.*)_unclassified", "Unclassified \\1"),
         Genus = str_replace_all(Genus,"_"," "))

gen_taxon_pool <- gen_rel_abund %>%
  dplyr::group_by(Genus) %>%
  dplyr::summarize(pool = mean(mean_rel_abund) < 0.58,
            med = mean(mean_rel_abund),
            .groups="drop")

gen <- dplyr::inner_join(gen_rel_abund, gen_taxon_pool, by="Genus") %>%
  dplyr::mutate(Genus= if_else(pool, "Other", Genus)) %>%
  dplyr::group_by(Disease_Timepoint, Genus, Disease_Status, Study_ID) %>%
  dplyr::filter(Genus != "Other")
#summarize(mean_rel_abund = sum(mean_rel_abund),
#          mean = min(mean),
#          .groups="drop")
library(ggprism)
genus_colors <- c("dodgerblue2", "goldenrod1", "dodgerblue2", "goldenrod1","dodgerblue2", "goldenrod1")
new_labels <- c("Alloprevotella"="*Alloprevotella*","Bacteroides"="*Bacteroides*","Bifidobacterium"="*Bifidobacterium*","Blautia"="*Blautia*","Butyricicoccus"="*Butyricicoccus*","Clostridium sensu stricto"="*Clostridium sensu stricto*","Collinsella"="*Collinsella*","Dorea"="*Dorea*","Escherichia/Shigella"="*Escherichia/Shigella*","Faecalibacterium"="*Faecalibacterium*","Fusobacterium"="*Fusobacterium*","Gallibacterium"="*Gallibacterium*","Mediterraneibacter"="*Mediterraneibacter*","Megamonas"="*Megamonas*","Phascolarctobacterium"="*Phascolarctobacterium*","Phocaeicola"="*Phocaeicola*","Prevotella"="*Prevotella*","Prevotellamassilia"="*Prevotellamassilia*","Streptococcus"="*Streptococcus*","Sutterella"="*Sutterella*","Unclassified Bacteroidaceae"="Uncl. *Bacteroidaceae*","Unclassified Bacteroidales"="Uncl. *Bacteroidales*","Unclassified Bacteroidetes"="Uncl. *Bacteroidetes*","Unclassified Clostridiales"="Uncl. *Clostridiales*","Unclassified Lachnospiraceae"="Uncl. *Lachnospiraceae*","Unclassified Muribaculaceae"="Uncl. *Muribaculaceae*","Unclassified Prevotellaceae"="Uncl. *Prevotellaceae*","Unclassified Ruminococcaceae"="Uncl. *Ruminococcaceae*","Unclassified Selenomonadaceae"="Uncl. *Selenomonadaceae*","Veillonella"="*Veillonella*")

ggplot(gen, aes(x = Disease_Timepoint, y = mean_rel_abund, fill=Disease_Timepoint)) +
  geom_boxplot(aes(fill = Disease_Timepoint, alpha = Disease_Timepoint), outlier.shape=NA) +
  facet_wrap(~Genus, ncol=5, labeller = labeller(Genus = new_labels)) +
  geom_jitter(width=0.15, aes(fill=Disease_Timepoint), alpha=0.5, color=1, pch=21, size=1) +
  geom_point(position = position_jitterdodge(jitter.width = 0.15), aes(fill=Disease_Timepoint), alpha=0.5, color=1, pch=21, size=1) +
  scale_x_discrete(breaks = c("Week 1 Control", "Week 1 Cryptosporidium_Positive","Week 5 Control", "Week 5 Cryptosporidium_Positive","Week 10 Control", "Week 10 Cryptosporidium_Positive"),
                   labels = c("H1<br>(n=33)", "*Cp*+1<br>(n=32)","H5<br>(n=32)", "*Cp*+5<br>(n=32)","H10<br>(n=32)", "*Cp*+10<br>(n=30)"),
                   drop = FALSE) +
  scale_y_continuous(expand = expansion(mult = c(0, 0.1)), limits = c(0,30)) +
  scale_fill_manual(values = genus_colors,
  name=NULL,
  breaks = c("Week 1 Control", "Week 1 Cryptosporidium_Positive","Week 5 Control", "Week 5 Cryptosporidium_Positive","Week 10 Control", "Week 10 Cryptosporidium_Positive"),
  labels = c("H1<br>(n=33)", "*Cp*+1<br>(n=32)","H5<br>(n=32)", "*Cp*+5<br>(n=32)","H10<br>(n=32)", "*Cp*+10<br>(n=30)")) +
  scale_alpha_manual(values=c(0.5,0.5,0.8,0.8,1,1),
  breaks = c("Week 1 Control", "Week 1 Cryptosporidium_Positive","Week 5 Control", "Week 5 Cryptosporidium_Positive","Week 10 Control", "Week 10 Cryptosporidium_Positive"),
  labels = c("H1<br>(n=33)", "*Cp*+1<br>(n=32)","H5<br>(n=32)", "*Cp*+5<br>(n=32)","H10<br>(n=32)", "*Cp*+10<br>(n=30)")) +
  labs(x = "", y = "Relative Abundance (%)") +
  stat_summary(fun = mean, geom = "point", color = "red")+
  theme_bw() +
  theme(axis.title.x = element_blank(),
        axis.text.x=element_markdown(size=5, face = "bold", colour = "black"),
        axis.text.y=element_text(colour="black"),
        legend.title = element_blank(),
        legend.text = element_markdown(),
        strip.text = element_markdown(size = 6),
        legend.position = "none") +
  guides(fill = guide_legend(nrow = 1))
```

Taxonomic MaAsLin2 Multivariate Analysis

```r
# Phylum OTU table
cdata_phylum <- calf_scaled %>%
  tax_glom(taxrank = "Phylum")
wholetax <- do.call(paste, c(as.data.frame(tax_table(cdata_phylum))[c("Phylum")],
    sep = "_")) # to distinguish from "_" within tax ranks
otu_phylum <- otu_table(cdata_phylum)
otu_phylum <- t(otu_phylum)
tmp <- taxa_names(otu_phylum)
# paste wholetax and OTU_ids together
for (i in 1:length(tmp)) {
    names(tmp)[i] = paste(tmp[i], wholetax[i], sep = "_")
}
taxa_names(otu_phylum) <- names(tmp)
head(otu_phylum)[5]

df_phylum <- as.data.frame(otu_phylum)
write.table(otu_phylum, file = "rare_otu_tab_phylum_names.txt",
    sep = "\t", quote = FALSE, row.names = TRUE, col.names = NA)

# All analysis was repeated with Farm 2 and Farm 3
# separately and together without Farm 1 to clarify the
# farm/antibiotic effect.

# Phylum Longitudinal and Pairwise Analysis
library(Maaslin2)
library(hilldiv)
library(tidyverse)
df_rare_phy = read.table(file = "rare_otu_tab_phylum_names.txt",
    header = TRUE, sep = "\t", row.names = 1, stringsAsFactors = FALSE,
    check.names = FALSE)
df <- t(df_rare_phy)
df <- tss(df)
phy_otu <- t(df)
phy_otu <- as.data.frame(phy_otu)

df_metadata = read.table(file = "16S_Metadata.txt", header = TRUE,
    row.names = 1, stringsAsFactors = FALSE, sep = "\t", check.names = FALSE)
df_metadata <- as.data.frame(df_metadata)

df_pair_W1 = df_metadata[df_metadata$Disease_Timepoint %in% c("Week 1 Control",
    "Week 1 Cryptosporidium_Positive"), ]
df_pair_W5 = df_metadata[df_metadata$Disease_Timepoint %in% c("Week 5 Control",
    "Week 5 Cryptosporidium_Positive"), ]
df_pair_W10 = df_metadata[df_metadata$Disease_Timepoint %in%
    c("Week 10 Control", "Week 10 Cryptosporidium_Positive"),
    ]

# Longitudinal Analysis
compare_all_phylum = Maaslin2(input_data = phy_otu, input_metadata = df_metadata,
    output = "16S_Maaslin2_Phylum_Day_of_Sampling_Analysis",
    fixed_effects = c("Disease_Status", "Farm", "Diatrim_1",
        "Diatrim_2", "Synulox", "Halocur", "Draaxin", "Day_of_Sampling"),
    random_effects = c("Study_ID"), normalization = "NONE", min_abundance = 1e-04,
    min_prevalence = 0.1, transform = "NONE", analysis_method = "LM",
    max_significance = 0.25, correction = "BH", standardize = TRUE,
    cores = 1, plot_heatmap = TRUE, plot_scatter = TRUE, heatmap_first_n = 50,
    reference = c("Farm,Farm 2"))

# Pairwise Comparisons
compare_W1_phylum = Maaslin2(input_data = phy_otu, input_metadata = df_pair_W1,
    output = "W1_16S_Maaslin2_Phylum_Analysis", fixed_effects = c("Disease_Status",
        "Farm", "Diatrim_1", "Synulox", "Halocur"), normalization = "NONE",
    min_abundance = 1e-04, min_prevalence = 0.1, transform = "NONE",
    analysis_method = "LM", max_significance = 0.25, correction = "BH",
    standardize = TRUE, cores = 1, plot_heatmap = TRUE, plot_scatter = TRUE,
    heatmap_first_n = 50, reference = c("Farm,Farm 2"))

compare_W5_phylum = Maaslin2(input_data = phy_otu, input_metadata = df_pair_W5,
    output = "W5_16S_Maaslin2_Phylum_Analysis", fixed_effects = c("Disease_Status",
        "Farm", "Diatrim_1", "Diatrim_2", "Synulox", "Halocur",
        "Draaxin"), normalization = "NONE", min_abundance = 1e-04,
    min_prevalence = 0.1, transform = "NONE", analysis_method = "LM",
    max_significance = 0.25, correction = "BH", standardize = TRUE,
    cores = 1, plot_heatmap = TRUE, plot_scatter = TRUE, heatmap_first_n = 50,
    reference = c("Farm,Farm 2"))

compare_W10_phylum = Maaslin2(input_data = phy_otu, input_metadata = df_pair_W10,
    output = "W10_16S_Maaslin2_Phylum_Analysis", fixed_effects = c("Disease_Status",
        "Farm", "Diatrim_1", "Diatrim_2", "Synulox", "Halocur",
        "Draaxin"), normalization = "NONE", min_abundance = 1e-04,
    min_prevalence = 0.1, transform = "NONE", analysis_method = "LM",
    max_significance = 0.25, correction = "BH", standardize = TRUE,
    cores = 1, plot_heatmap = TRUE, plot_scatter = TRUE, heatmap_first_n = 50,
    reference = c("Farm,Farm 2"))

# Combined results tables from each of these runs into .txt
# file: all_results_phylum_pairwise.txt

# Recalculate q-values after pairwise comparisons
maaslin2_all_results <- read_delim(file = "all_results_phylum_pairwise.txt")
maaslin2_all_results$recalc_qval <- p.adjust(maaslin2_all_results$pval,
    method = "BH")

# Genus OTU Table
cdata_genus <- calf_scaled %>%
    tax_glom(taxrank = "Genus")
wholetax <- do.call(paste, c(as.data.frame(tax_table(cdata_genus))[c("Genus")],
    sep = "_")) # to distinguish from "_" within tax ranks
otu_genus <- otu_table(cdata_genus)
otu_genus <- t(otu_genus)
tmp <- taxa_names(otu_genus)
# paste wholetax and OTU_ids together
for (i in 1:length(tmp)) {
    names(tmp)[i] = paste(tmp[i], wholetax[i], sep = "_")
}
taxa_names(otu_genus) <- names(tmp)
head(otu_genus)[5]

df_genus <- as.data.frame(otu_genus)
# colnames(df_genus) <-
# as.data.frame(tax_table(cdata_genus))$Genus
write.table(df_genus, file = "rare_otu_tab_genus_names.txt",
    sep = "\t", quote = FALSE, row.names = TRUE, col.names = NA)

# All analysis was repeated with Farm 2 and Farm 3
# separately and together without Farm 1 to clarify the
# farm/antibiotic effect.

# Genus Longitudinal and Pairwise Analysis
df_rare_gen = read.delim(file = "rare_otu_tab_genus_names.txt",
    header = TRUE, sep = "\t", row.names = 1, stringsAsFactors = FALSE,
    check.names = FALSE)
df <- t(df_rare_gen)
df <- tss(df)
gen_otu <- t(df)
gen_otu <- as.data.frame(gen_otu)

df_metadata = read.table(file = "16S_Metadata.txt", header = TRUE,
    sep = "\t", row.names = 1, stringsAsFactors = FALSE)

# Longitudinal Analysis
compare_all_genus = Maaslin2(input_data = gen_otu, input_metadata = df_metadata,
    output = "16S_Maaslin2_Genus_Day_of_Sampling_Analysis", fixed_effects = c("Disease_Status",
        "Farm", "Diatrim_1", "Diatrim_2", "Synulox", "Draaxin",
        "Halocur", "Day_of_Sampling"), random_effects = c("Study_ID"),
    normalization = "NONE", min_abundance = 1e-04, min_prevalence = 0.1,
    transform = "NONE", analysis_method = "LM", max_significance = 0.25,
    correction = "BH", standardize = TRUE, cores = 1, plot_heatmap = TRUE,
    plot_scatter = TRUE, heatmap_first_n = 50, reference = c("Farm,Farm 2"))

# Pairwise Comparisons
df_pair_W1 = df_metadata[df_metadata$Disease_Timepoint %in% c("Week 1 Control",
    "Week 1 Cryptosporidium-Positive"), ]
df_pair_W5 = df_metadata[df_metadata$Disease_Timepoint %in% c("Week 5 Control",
    "Week 5 Cryptosporidium-Positive"), ]
df_pair_W10 = df_metadata[df_metadata$Disease_Timepoint %in%
    c("Week 10 Control", "Week 10 Cryptosporidium-Positive"),
    ]

compare_W1_genus = Maaslin2(input_data = gen_otu, input_metadata = df_pair_W1,
    output = "W1_16S_Maaslin2_Genus_Analysis", fixed_effects = c("Disease_Status",
        "Farm", "Diatrim_1", "Synulox", "Halocur"), normalization = "NONE",
    min_abundance = 1e-04, min_prevalence = 0.1, transform = "NONE",
    analysis_method = "LM", max_significance = 0.25, correction = "BH",
    standardize = TRUE, cores = 1, plot_heatmap = TRUE, plot_scatter = TRUE,
    heatmap_first_n = 200, reference = c("Farm,Farm 2"))

compare_W5_genus = Maaslin2(input_data = gen_otu, input_metadata = df_pair_W5,
    output = "W5_16S_Maaslin2_Genus_Analysis", fixed_effects = c("Disease_Status",
        "Farm", "Diatrim_1", "Diatrim_2", "Synulox", "Halocur",
        "Draaxin"), normalization = "NONE", min_abundance = 1e-04,
    min_prevalence = 0.1, transform = "NONE", analysis_method = "LM",
    max_significance = 0.25, correction = "BH", standardize = TRUE,
    cores = 1, plot_heatmap = TRUE, plot_scatter = TRUE, heatmap_first_n = 200,
    reference = c("Farm,Farm 2"))

compare_W10_genus = Maaslin2(input_data = gen_otu, input_metadata = df_pair_W10,
    output = "W10_16S_Maaslin2_Genus_Analysis", fixed_effects = c("Disease_Status",
        "Farm", "Diatrim_1", "Diatrim_2", "Synulox", "Halocur",
        "Draaxin"), normalization = "NONE", min_abundance = 1e-04,
    min_prevalence = 0.1, transform = "NONE", analysis_method = "LM",
    max_significance = 0.25, correction = "BH", standardize = TRUE,
    cores = 1, plot_heatmap = TRUE, plot_scatter = TRUE, heatmap_first_n = 200,
    reference = c("Farm,Farm 2"))

# Combined results tables from each of these runs into .txt
# file: all_results_genus_pairwise.txt

# Recalculate q-values after pairwise comparisons
maaslin2_all_results <- read_delim(file = "all_results_genus_pairwise.txt")
maaslin2_all_results$qval <- p.adjust(maaslin2_all_results$pval,
    method = "BH")
```

Body Condition Score, Daily Live Weight Gain, and Respiratory Disease Scoring Analysis

```r
library(ggplot2)
library(ggpubr)
library(tidyverse)
library(scales)
library(vegan)
library(ape)
library(phyloseq)
library(ggtext)
library(ggpmisc)

# Figure 5A - Body Condition Score (BCS)
metadata_BCS <- read_delim("16S_Metadata.txt")
metadata_BCS$Disease_Timepoint <- factor(metadata_BCS$Disease_Timepoint,
    levels = c("Week 1 Control", "Week 1 Cryptosporidium_Positive",
        "Week 5 Control", "Week 5 Cryptosporidium_Positive",
        "Week 10 Control", "Week 10 Cryptosporidium_Positive"))
shapiro.test(metadata_BCS$Body_Condition_Score)
cm_bcs <- compare_means(Body_Condition_Score ~ Disease_Timepoint,
    metadata_BCS, method = "wilcox.test", size = 3, p.adjust.method = "BH")
cm_bcs <- cm_bcs[!(row.names(cm_bcs) %in% c("2", "3", "4", "5",
    "6", "7", "8", "9", "11", "12", "13", "14")), ]

ggplot(metadata_BCS, aes(x = Disease_Timepoint, y = Body_Condition_Score,
    fill = Disease_Timepoint)) + geom_boxplot(aes(alpha = Disease_Timepoint)) +
    geom_point(aes(fill = Disease_Timepoint), alpha = 0.5, color = 1,
        pch = 21, size = 3, position = position_jitter(width = 0.2,
            height = 0)) + scale_fill_manual(values = c("dodgerblue2",
    "goldenrod1", "dodgerblue2", "goldenrod1", "dodgerblue2",
    "goldenrod1"), breaks = c("Week 1 Control", "Week 1 Cryptosporidium_Positive",
    "Week 5 Control", "Week 5 Cryptosporidium_Positive", "Week 10 Control",
    "Week 10 Cryptosporidium_Positive")) + scale_color_manual(values = c("dodgerblue2",
    "goldenrod1", "dodgerblue2", "goldenrod1", "dodgerblue2",
    "goldenrod1"), breaks = c("Week 1 Control", "Week 1 Cryptosporidium_Positive",
    "Week 5 Control", "Week 5 Cryptosporidium_Positive", "Week 10 Control",
    "Week 10 Cryptosporidium_Positive")) + scale_x_discrete(labels = c("H1<br>(n=33)",
    "*Cp*+1<br>(n=32)", "H5<br>(n=32)", "*Cp*+5<br>(n=31)", "H10<br>(n=32)",
    "*Cp*+10<br>(n=30)")) + scale_alpha_manual(values = c(0.5,
    0.5, 0.8, 0.8, 1, 1)) + stat_summary(fun = mean, geom = "point",
    color = "red") + labs(x = "", y = "Body Condition Score",
    tag = "A") + theme_classic() + theme(legend.position = "none",
    text = element_text(colour = "black", size = 10), axis.text.x = element_markdown(colour = "black"),
    axis.text.y = element_text(colour = "black")) + stat_pvalue_manual(cm_bcs,
    label = "p.adj", y.position = 3.6, step.increase = 0, inherit.aes = FALSE,
    size = 3)

# Figure 5B - Daily Live Weight Gain (Farm 2)
bwm <- read_delim("16S_Metadata.txt")
bwm = bwm[bwm$Farm %in% c("Farm 2"), ]
df_Farm2 = bwm[bwm$Disease_Timepoint %in% c("Week 5 Cryptosporidium_Positive",
    "Week 5 Control", "Week 10 Control", "Week 10 Cryptosporidium_Positive"),
    ]
df_Farm2$Disease_Timepoint <- factor(df_Farm2$Disease_Timepoint,
    levels = c("Week 1 Control", "Week 1 Cryptosporidium_Positive",
        "Week 5 Control", "Week 5 Cryptosporidium_Positive",
        "Week 10 Control", "Week 10 Cryptosporidium_Positive"))

shapiro.test(df_Farm2$DLWG_kg)
cm_dlwg <- compare_means(DLWG_kg ~ Disease_Timepoint, df_Farm2,
    method = "t.test", size = 3, p.adjust.method = "BH")
cm_dlwg <- cm_dlwg[!(row.names(cm_dlwg) %in% c("2", "3", "4",
    "5")), ]

ggplot(df_Farm2, aes(x = DLWG_kg)) + geom_histogram(binwidth = 0.1,
    color = "black", fill = "dodgerblue2") + theme_classic()

ggplot(df_Farm2, aes(x = Disease_Timepoint, y = DLWG_kg, fill = Disease_Timepoint)) +
    geom_boxplot(aes(alpha = Disease_Timepoint)) + geom_point(aes(fill = Disease_Timepoint),
    alpha = 0.5, color = 1, pch = 21, size = 3) + scale_fill_manual(values = c("dodgerblue2",
    "goldenrod1", "dodgerblue2", "goldenrod1"), breaks = c("Week 5 Control",
    "Week 5 Cryptosporidium_Positive", "Week 10 Control", "Week 10 Cryptosporidium_Positive")) +
    scale_color_manual(values = c("dodgerblue2", "goldenrod1",
        "dodgerblue2", "goldenrod1"), breaks = c("Week 5 Control",
        "Week 5 Cryptosporidium_Positive", "Week 10 Control",
        "Week 10 Cryptosporidium_Positive")) + scale_x_discrete(labels = c("H5<br>(n=17)",
    "*Cp*+5<br>(n=20)", "H10<br>(n=20)", "*Cp*+10<br>(n=22)")) +
    ylim(-0.05, 1.5) + scale_alpha_manual(values = c(0.8, 0.8,
    1, 1)) + stat_summary(fun = mean, geom = "point", color = "red") +
    labs(x = "", y = bquote("Daily Live Weight Gain" ~ (kg ~
        day^-1)), tag = "A") + theme_classic() + theme(legend.position = "none",
    text = element_text(colour = "black", size = 10), axis.text.x = element_markdown(colour = "black"),
    axis.text.y = element_text(colour = "black")) + stat_pvalue_manual(cm_dlwg,
    label = "p.adj", y.position = 0.9, step.increase = 0.35,
    inherit.aes = FALSE, size = 3)

# Figure 5C - WI Total Score
wim <- read_delim("16S_Metadata.txt")
wim$Disease_Timepoint <- factor(wim$Disease_Timepoint, levels = c("Week 1 Control",
    "Week 1 Cryptosporidium_Positive", "Week 5 Control", "Week 5 Cryptosporidium_Positive",
    "Week 10 Control", "Week 10 Cryptosporidium_Positive"))

shapiro.test(wim$Total_WI_Score)
cm_wim <- compare_means(Total_WI_Score ~ Disease_Timepoint, wim,
    method = "wilcox.test", size = 3, p.adjust.method = "BH")
cm_wim <- cm_wim[!(row.names(cm_wim) %in% c("2", "3", "4", "5",
    "6", "7", "8", "9", "11", "12", "13", "14")), ]

ggplot(wim, aes(x = Disease_Timepoint, y = Total_WI_Score, fill = Disease_Timepoint)) +
    geom_boxplot() + geom_point(aes(fill = Disease_Timepoint),
    alpha = 0.5, color = 1, pch = 21, size = 3, position = position_jitter(width = 0.35,
        height = 0)) + scale_y_continuous(breaks = seq(0, 6,
    by = 1)) + scale_fill_manual(values = c("dodgerblue2", "goldenrod1",
    "dodgerblue2", "goldenrod1", "dodgerblue2", "goldenrod1"),
    breaks = c("Week 1 Control", "Week 1 Cryptosporidium_Positive",
        "Week 5 Control", "Week 5 Cryptosporidium_Positive",
        "Week 10 Control", "Week 10 Cryptosporidium_Positive")) +
    scale_color_manual(values = c("dodgerblue2", "goldenrod1",
        "dodgerblue2", "goldenrod1", "dodgerblue2", "goldenrod1"),
        breaks = c("Week 1 Control", "Week 1 Cryptosporidium_Positive",
            "Week 5 Control", "Week 5 Cryptosporidium_Positive",
            "Week 10 Control", "Week 10 Cryptosporidium_Positive")) +
    scale_x_discrete(labels = c("H1<br>(n=33)", "*Cp*+1<br>(n=32)",
        "H5<br>(n=32)", "*Cp*+5<br>(n=32)", "H10<br>(n=32)",
        "*Cp*+10<br>(n=30)")) + scale_alpha_manual(values = c(0.5,
    0.5, 0.8, 0.8, 1, 1)) + labs(x = "", y = "Total WI Score",
    tag = "B") + stat_summary(fun = mean, geom = "point", color = "red") +
    theme_classic() + theme(legend.position = "none", text = element_text(colour = "black",
    size = 10), axis.text.x = element_markdown(colour = "black"),
    axis.text.y = element_text(colour = "black")) + stat_pvalue_manual(cm_wim,
    label = "p.adj", y.position = 5, step.increase = 0.1, inherit.aes = FALSE,
    size = 3)

# Figure 5D - W10 Lung Scores
LS_wim = wim[wim$Disease_Timepoint %in% c("Week 10 Control",
    "Week 10 Cryptosporidium_Positive"), ]

shapiro.test(LS_wim$Lung_Score)
cm_ls <- compare_means(Lung_Score ~ Disease_Status, LS_wim, method = "wilcox.test",
    size = 3, p.adjust.method = "BH")

ggplot(LS_wim, aes(y = Lung_Score, x = Disease_Status, fill = Disease_Status)) +
    geom_boxplot() + geom_point(aes(fill = Disease_Status), alpha = 0.5,
    color = 1, pch = 21, size = 3, position = position_jitter(width = 0.25,
        height = 0)) + scale_y_continuous(breaks = seq(0, 5,
    by = 1)) + scale_fill_manual(values = c("dodgerblue2", "goldenrod1"),
    breaks = c("Control", "Cryptosporidium_Positive")) + scale_color_manual(values = c("dodgerblue2",
    "goldenrod1"), breaks = c("Control", "Cryptosporidium_Positive")) +
    scale_x_discrete(labels = c("H10<br>(n=30)", "*Cp*+10<br>(n=30)")) +
    labs(y = "Lung Score", x = "", tag = "C") + stat_summary(fun = mean,
    geom = "point", color = "red") + theme_classic() + theme(legend.position = "none",
    text = element_text(colour = "black", size = 10), axis.text.x = element_markdown(colour = "black"),
    axis.text.y = element_text(colour = "black")) + stat_pvalue_manual(cm_ls,
    label = "p.format", size = 3, y.position = 5.5, inherit.aes = FALSE)
```
